# Impact of lignin depolymerization on aerobic and anaerobic bioconversion of alkaline liquor from sugarcane bagasse

**DOI:** 10.3389/fbioe.2026.1837262

**Published:** 2026-07-14

**Authors:** Fabrícia Farias de Menezes, Fernanda Miyuki Kashiwagi, Jessica Jacinta Silva, Gustavo Rodrigues Gomes, Rafaela Prata, Maria Rosa de Moraes, Adriano Freitas Lima, Felipe Garcia da Silva, Renata Piacentini Rodriguez, Carlos Eduardo Driemeier, George Jackson de Moraes Rocha, Priscila Oliveira de Giuseppe

**Affiliations:** 1 Brazilian Biorenewables National Laboratory (LNBR), Brazilian Center for Research in Energy and Materials (CNPEM), Campinas, São Paulo, Brazil; 2 Institute of Science and Technology (ICT), Federal University of Alfenas (UNIFAL), Poços de Caldas, Minas Gerais, Brazil

**Keywords:** aerobic bioconversion, alkaline liquor, anaerobic digestion, aromatic, biogas, biomethane, lignin depolymerization

## Abstract

**Introduction:**

Lignin recalcitrance, together with its complex interactions with cellulose and hemicelluloses within the plant cell wall, remains a major barrier to efficient lignocellulosic biomass valorization. Mild alkaline pretreatment partially addresses this challenge by solubilizing lignin and acetate into a lignin-rich alkaline liquor (AL). However, the recovered lignin is largely oligomeric, limiting its direct microbial conversion.

**Methods:**

To evaluate strategies for improving AL bioconversion, alkaline liquor was subjected to thermochemical depolymerization under different severities, generating liquors with compositions ranging from oligomer-rich to monomer-rich profiles. The resulting streams were evaluated through two bioconversion routes: aerobic metabolism by *Pseudomonas putida* KT2440 and anaerobic digestion for biomethane production.

**Results:**

Mild to moderate depolymerization conditions (≤240 °C) improved the growth of *P. putida*, whereas the highest depolymerization severity strongly inhibited bacterial growth. This inhibition was associated with increased concentrations of aromatic monomers that are poorly metabolized by *P. putida*, including phenol and alkyl-substituted phenols. In anaerobic digestion assays, mild to moderate depolymerization yielded the highest specific methane productions, corresponding to a 10%–20% increase relative to non-depolymerized AL, while severe depolymerization conditions negatively affected methane production.

**Discussion:**

These findings demonstrate that controlled lignin depolymerization can enhance both aerobic and anaerobic bioconversion by balancing chemical accessibility with biological compatibility. This approach provides a promising strategy for improving lignin utilization within integrated biorefinery concepts.

## Graphical Abstract

**Figure:**
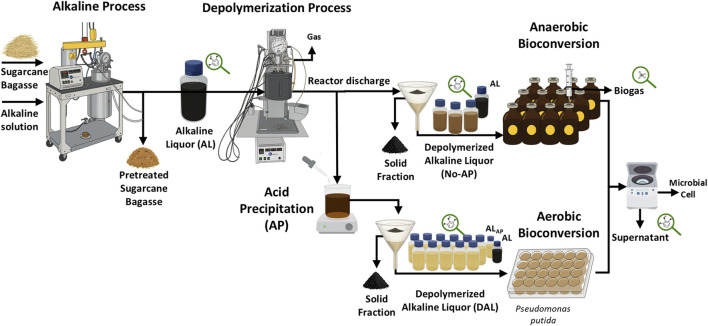

## 1 Introduction

Lignocellulosic biomass, an abundant and renewable resource, holds significant potential for conversion into valuable products such as biofuels and biochemicals (Ashokkumar et al., 2022). Among the various types of feedstocks, sugarcane bagasse stands out due to its high availability at the processing site, especially in countries with extensive sugarcane production and biorefining such as Brazil (Freitas et al., 2021). However, the efficient conversion of lignocellulosic biomass is challenging due to its recalcitrant nature (Yoo et al., 2020). To decrease this recalcitrance, physicochemical processes are usually employed, combining different conditions of temperature, pressure and pH (Chaudhary et al., 2024).

Alkaline processes under mild conditions (typically <100 °C and atmospheric pressure) have emerged as an attractive alternative to conventional high-temperature and high-pressure acidic pretreatments (Schutyser et al., 2018; Kuhn et al., 2020; Cedeno et al., 2026). Although they present challenges related to alkali recovery, effluent management, and overall process sustainability (Ghosh et al., 2025), these alkaline approaches promote the solubilization of lignin and acetyl groups in the liquid fraction while preserving polysaccharides in the solid fraction, potentially reducing operational costs and minimizing the formation of inhibitory compounds that hinder cellulose hydrolysis and subsequent glucose fermentation (Davis et al., 2018; Kuhn et al., 2020). Compared with other emerging environmentally friendly alternatives, mild alkaline processes offer practical advantages, including shorter processing times relative to biological pretreatments, as well as higher fermentative compatibility and lower costs relative to ionic liquid-based systems (Yin et al., 2021).

Lignin is the most abundant renewable source of aromatic compounds and plays a crucial role in the transition from fossil-based industries to sustainable, bio-based technologies (Menezes et al., 2023). Lignin depolymerization aims to convert its high-molecular-weight structure into lower molecular weight fractions, producing streams enriched in monomeric aromatic compounds. These fractions can be upgraded to produce bioproducts such as renewable chemicals (Silva et al., 2025), liquid (Kariim et al., 2025), and gaseous biofuels (Mulat and Horn, 2018). However, studies demonstrating the potential of lignin-derived streams to be used for biogas production are scarce and are usually limited to assays using model compounds to understand toxicity effects (Barakat et al., 2012; Wu and He, 2013; Monlau et al., 2014; Radhika et al., 2021; Li et al., 2025; Sun et al., 2024).

Bio-based upgrading of lignin-derived streams can be a good alternative to valorize them (Linger et al., 2014; Chen et al., 2024). Some lignin-derived monomers present in alkaline liquors from softwood (Suzuki et al., 2021), corn stover (Zong et al., 2022), and sugarcane bagasse (Akutsu et al., 2022) have been demonstrated to be compatible with aerobic bioconversion pathways aiming to produce valuable chemicals such as beta-ketoadipate, polyhydroxyalkanoates, and muconic acid. However, these studies focused on the monomeric aromatic compounds observed in alkaline liquor, overlooking acetate, and macromolecular lignin, which partly precipitates when the liquor pH is lowered. In this context, *P. putida* KT2440 can serve as an ideal bacterial chassis for investigating the co-valorization of acetate and aromatic compounds. This strain naturally metabolizes acetate and harbors multiple pathways (Weiland et al., 2022) that funnel a wide range of lignin-derived aromatic compounds into central metabolites, particularly acetyl-CoA, which represents a key metabolic node shared by both acetate utilization and aromatic catabolism. Moreover, *Pseudomonas putida* KT2440 is a safe strain, with a versatile metabolism, resistant to harsh conditions, and which benefits from a well-developed set of genetic engineering tools, rendering it an ideal chassis for biotechnological applications (Nelson et al., 2002; de Lorenzo et al., 2024; Werner et al., 2023).

Anaerobic digestion is another promising bioconversion approach for lignin valorization. In the sugarcane industry, anaerobic digestion has been scaled commercially and primarily targets residues like vinasse and filter cake from 1G ethanol production. Integrating the processing of sugarcane bagasse into second-generation (2G) biorefineries introduces new potential streams for anaerobic digestion, opening opportunities to enhance efficiency and address challenges associated with seasonal operation. Recent studies have demonstrated the feasibility of digesting alkaline liquors rich in lignin alone or with vinasse or filter cake from 1G production ​(Volpi et al., 2022; 2023). However, it is still not well understood which lignin-derived compounds can be effectively metabolized under anaerobic conditions, nor how their molecular characteristics, such as molecular weight and functionalization, influence their conversion into biogas.

Submitting alkaline liquor directly to lignin depolymerization reactions, prior to the biological upgrading step, could be advantageous in terms of operational efficiency. Processing the liquor would leverage that lignin is already solubilized in an alkaline solution, with NaOH and water actively participating in bond-cleavage reactions (Lappalainen et al., 2020). However, the potential of this simplified process to improve the aerobic bioconversion efficiency of alkaline liquors has not yet been demonstrated. Another unresolved question is whether submitting alkaline liquors to lignin depolymerization reactions would improve their conversion into biogas via anaerobic digestion. Studies on the anaerobic digestion of isolated lignin-derived streams remain scarce ​(Radhika et al., 2021; Sun et al., 2024; Cedeno et al., 2026) and the slow rate of biological lignin depolymerization has been identified as a limiting factor for the industrial applicability of anaerobic digestion in lignin valorization (Mulat and Horn, 2018).

In this study, we hypothesized that depolymerization severity modulates the microbial conversion efficiency of lignin solubilized by mild alkaline pretreatment. To test this, a lignin-rich alkaline liquor obtained from sugarcane bagasse was subjected to thermochemical depolymerization under systematically varied conditions of temperature, reaction time, and O_2_ pressure. This approach generated liquors with distinct molecular weight distributions and compositional profiles of lignin-derived compounds. These depolymerized liquors were evaluated as substrates for (i) aerobic conversion using *P*. *putida* KT2440, and (ii) anaerobic digestion for methane production using anaerobic sludge as inoculum. *Pseudomonas putida* KT2440 was chosen as a model for aerobic conversion due to the extensive research using this microorganism as a biofactory to convert lignin-derived compounds into value-added molecules (Zhu et al., 2025) whereas an anaerobic sludge from a consolidated agro-industrial facility was selected to evaluate methane production due to its scalability. By integrating quantitative chemical characterization with microbial growth and conversion assays, this study reveals how lignin depolymerization shapes the bioconversion potential of alkaline liquors, providing guidance for the development of efficient and sustainable lignin valorization strategies within biorefineries.

## 2 Methodology

**FIGURE 1:**
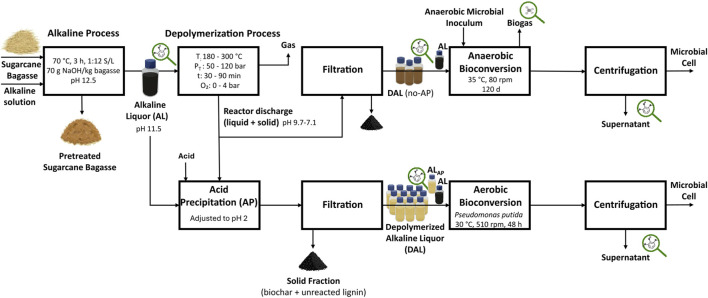
Methodology overview. Sugarcane bagasse underwent alkaline pretreatment to produce AL. Subsequently, AL was subjected to hydrothermal process under varying experimental conditions, including temperature and pressure, reaction time, and oxygen addition, resulting in 11 distinct DALs (Table 1). After acid precipitation, AL_AP_ and DALs were tested in aerobic conversion assays. AL without acid precipitation was also tested in aerobic conversion as a control. For anaerobic conversion, selected liquors (AL, DAL_1_, DAL_5_, DAL_11_) were used directly, without acid precipitation. AL, DALs, supernatants, and biogas were subsequently characterized (indicated by the magnifying glass symbol).

This study aimed to evaluate two bioconversion routes using alkaline liquor (AL) and depolymerized alkaline liquors (DALs) as the main carbon source. The aerobic route assessed the metabolism of aromatic compounds and aliphatic acids by *P. putida* KT2440 (ATCC 47054), while the anaerobic route targeted biogas production through biochemical methane potential (BMP) assays using an inoculum from a mesophilic upflow anaerobic sludge blanket (UASB) reactor treating poultry slaughterhouse wastewater (Figure 1).

### 2.1 Alkaline liquor (AL) production

To produce an alkaline liquor (AL) suitable for subsequent depolymerization and bioconversion studies, sugarcane bagasse (∼12% moisture), previously ground to ≤6 mm using a cross-beater mill (Retsch SK 100), was obtained from a sugarcane mill in São Paulo State (harvested in October 2022). The alkaline treatment was carried out in a 7.5 L Parr reactor (model 4,848) at 70 °C for 3 h, using a solid-to-liquid ratio of 1:12 (bagasse:alkaline solution) and a NaOH loading of 70 g kg^-1^ bagasse (pH 12.5) (Cedeno et al., 2026). After the reaction, the supernatant was filtered, and the liquid fraction (alkaline liquor, AL) was collected for subsequent depolymerization reactions.

For chromatographic analysis (HPLC and GC-MS), an aliquot of AL was subjected to acid precipitation (AP) by adjusting the pH to 2 with 72% (w/w) H_2_SO_4_. The liquid fraction obtained after acid precipitation was hereafter referred to as AL_AP_. Both AL and AL_AP_ were subsequently submitted to analyses described in Section 2.5.

### 2.2 Depolymerized alkaline liquors (DALs) production

To generate depolymerized alkaline liquors (DALs) for bioconversion studies, alkaline liquor (AL) from sugarcane bagasse was subjected to hydrothermal depolymerization following a 2^3^ factorial design (8 experimental runs and 1 central point in triplicate). Reactions were performed varying temperature (180 °C–300 °C), reaction time (30–90 min), and O_2_ addition (0–4 bar) (Menezes et al., 2022). The addition of O_2_ (4 bar) aimed to investigate whether a mild oxidative environment would increase the yield of oxidized aromatic monomers while avoiding extensive ring-opening reactions, potentially improving substrate biocompatibility for subsequent bioconversion. Experiments were conducted in a high-pressure batch reactor (500 mL, model 4575A, Parr Instrument Company), with precise control of temperature, pressure, and agitation (Parr 4,848). The total pressures reached approximately 50 bars at 180 °C, 70 bars at 240 °C, and 120 bars at 300 °C. The conditions for all 11 depolymerization reactions are summarized in Table 1.

**TABLE 1:** Experimental conditions (temperature, time, and initial O_2_ added to the reaction) for the 11 depolymerization reactions that generated different Depolymerized Alkaline Liquors (DALs).

T (°C)	Time (min)	O_2_ addition (bar)	Depolymerized liquor	Bioconversion assay
180	30	0	DAL_1_	Aerobic and anaerobic
180	30	4	DAL_2_	Aerobic
180	90	0	DAL_3_	Aerobic
180	90	4	DAL_4_	Aerobic
240	60	2	DAL_5_	Aerobic and anaerobic
240	60	2	DAL_6_	Aerobic
240	60	2	DAL_7_	Aerobic
300	30	0	DAL_8_	Aerobic
300	30	4	DAL_9_	Aerobic
300	90	0	DAL_10_	Aerobic
300	90	4	DAL_11_	Aerobic and anaerobic

Each reaction was initiated with 250 g of alkaline liquor (AL). The reactor, sealed with a graphite gasket, was purged three times with nitrogen (N_2_), pressurized to 26–30 bar with N_2_, and subsequently adjusted with oxygen (O_2_, addition of 0–4 bar) to reach the desired final pressure of 30 bar. Agitation was maintained at 500 rpm throughout the entire process. After completion of the reaction, the mixture was processed according to the requirements of the subsequent biological assays.

For aerobic assays, the pH was adjusted to 2 with 72% sulfuric acid to precipitate macromolecular lignin (Hubbe et al., 2019), and the liquid fraction (DALs) was recovered by filtration. For comparison purposes, the same procedure was applied to an AL aliquot, generating the AL_AP_ sample. Both AL and AL_AP_ samples were submitted to aerobic growth assays along with the DALs samples (Section 2.3). Acid precipitation was applied to remove macromolecular lignin prior to the aerobic assays, enriching the liquors in monomeric compounds, as *P. putida* KT2440 is known to primarily metabolize aromatic monomers (Lee et al., 2023).

For anaerobic assays, in contrast, no acid precipitation was performed; only a filtration step was applied to remove dispersed solids. As the complex microbial consortium involved in anaerobic digestion exhibits diverse metabolic capabilities, we hypothesized that it would be capable of metabolizing not only monomeric species but also higher-molecular-weight aromatic compounds.

### 2.3 Aerobic bioconversion

*Pseudomonas putida* KT2440 (ATCC 47054) was used to test the bioconversion of the AL, AL_AP_ and DAL_1_–DAL_11_, serving as a model for aerobic routes. Single colonies of *P. putida* were inoculated and grown on liquid LB medium (10 g L^-1^ peptone, 5 g L^-1^ yeast extract and 10 g L^-1^ NaCl) at 30 °C, 200 rpm. Cells were washed twice with XVM2 minimal medium (20 mM NaCl, 10 mM (NH_4_)_2_SO_4_, 1 mM CaCl_2_, 0.01 mM FeSO_4_.7H_2_O, 5 mM MgSO_4_, 0.16 mM KH_2_PO_4_, 0.32 mM K_2_HPO_4_, 0.3 g L^-1^ casamino acids) and inoculated to an initial optical density at 600 nm (OD_600_) of 0.05 on XVM2 medium supplemented with the liquors. Prior to inoculation, the liquors were adjusted to pH 6.7 and filter sterilized. For each assay, the liquors (AL, AL_AP_ and DAL_1_–DAL_11_) were diluted as necessary to achieve a standardized concentration of 1 g L^-1^ of total soluble aromatics, as estimated by UV-vis spectroscopy (Section 2.5.1), in an attempt to minimize the effect of variations of total aromatics loading on microbial growth.

The cultures were performed in 24-well plates with incubation and simultaneous OD_600_ measurement in the Tecan Spark Microplate Multimode Reader or Tecan Infinite M200 Pro at 30 °C and orbital shaking of 510 rpm. Growth package was used to calculate the specific growth rates and lag time. After 48 h of culturing, cells were harvested and the supernatant was collected for measurement of aromatic compounds and organic acids by GC-MS and HPLC, as described in the 2.5 characterization section.

### 2.4 Anaerobic bioconversion

The non-depolymerized liquor (AL) and three representative depolymerized liquors (DAL_1_, DAL_5_, and DAL_11_) were directly used for biogas production, without acid precipitation. The inoculum used in the biochemical methane potential (BMP) assays was obtained from a mesophilic upflow anaerobic sludge blanket (UASB) reactor treating poultry slaughterhouse wastewater (Pereiras, São Paulo State, Brazil). The three liquors (DAL1, DAL5, and DAL11) were selected to represent distinct levels of depolymerization severity within the experimental design, corresponding to mild (DAL1), moderate (DAL5), and severe (DAL11) conditions.

Biogas and methane production from alkaline liquor and depolymerized liquors were evaluated using biochemical methane potential (BMP) assays in mono-digestion, where each liquor served as the sole substrate, following the VDI 4630 methodology (Verein Deutscher Ingenieure, 2006)​, which recommends a substrate-to-inoculum ratio of 1:2 based on total volatile solids (TVS).

Experiments were conducted in 120 mL borosilicate glass bottles with a working volume of 70 mL (50 mL headspace), incubated at 35 °C and 80 rpm. The volumes of substrate and inoculum were adjusted for each liquor to comply with a TVS-based substrate-to-inoculum ratio of 1:2, as follows: 51 mL AL + 19 mL inoculum; 54 mL DAL_1_ + 16 mL inoculum; 56 mL DAL_5_ + 14 mL inoculum; and 58 mL DAL_11_ + 12 mL inoculum. The TVS values for the liquors and inoculum are reported in Section 3.3. Negative controls containing only inoculum were included. The pH was adjusted to 7.0 using 1 M HCl.

Cumulative biogas production was measured using a 60 mL hypodermic syringe (Descarpack) until the biogas volume varied by less than 1% over three consecutive measurements. All assays were performed in triplicate, and standard deviation was used as the error bar for statistical analysis.

Chemical oxygen demand (COD) and solids content were determined according to Standard Methods for the Examination of Water and Wastewater (APHA, 2023)​. Biogas composition was analyzed following ​Volpi et al. (2022)​. The kinetics of cumulative methane production under each experimental condition were analyzed using the modified Gompertz equation (Equation 1) to estimate methane production potential, maximum methane production rate, and lag phase. Model fitting was performed using OriginPro 2025b. The goodness-of-fit was evaluated using the coefficient of determination (R^2^), residual sum of squares (RSS), and root mean square error (RMSE).

Equation 1:

$$P_{{CH}_{4}}\left(t\right)=P_{\max}*\exp\;\left(-\exp\;\left(\left(\frac{R_{\max}*\;e}{P_{\max}}\right)*\left(\lambda -t\right)+1\;\right)\right)$$ (1)

Where:

P_CH4_ (t): cumulative production of methane (NmL CH_4_) over time. P_max_: maximum methane production potential (NmL CH_4_). R_max_: maximum methane production rate (NmL CH_4_ d^-1^). λ: lag phase (d).

The specific methane production was expressed in NmL CH_4_ g^-1^ VS, corresponding to the normalized methane volume per Gram of volatile solids added. At the end of the experiments, the assays were harvested, and the supernatants were collected for quantification of aromatic compounds and organic acids by GC–MS and HPLC, as described in Section 2.5.

### 2.5 Characterization

#### 2.5.1 UV–vis spectroscopy

The concentration of total soluble aromatic compounds in AL and DAL liquors was inferred by UV–vis spectroscopy at 280 nm using a Thermo Scientific Evolution 300 spectrophotometer, according to Equation 2 (Rocha et al., 2015; Sluiter et al., 2016).

Equation 2:

$$C=\frac{A_{280\;nm}\;*\;f_{d\;}-B}{A}$$ (2)

Where: *C* is the concentration of total soluble aromatic compounds in the liquors (g L^-1^); A_280nm_ is the absorbance at 280 nm; *f*_*d*_ is the dilution factor; *B* is the linear coefficient for sugarcane bagasse lignin (0.018, experimental value); and *A* is the angular coefficient corresponding to the absorptivity of sugarcane bagasse lignin (23.7 L g^-1^ cm^-1^, experimental value). Of note, this estimative must be interpreted as an apparent concentration as it does not account for potential changes in absorptivity resulting from lignin fragmentation.

#### 2.5.2 High–performance liquid chromatography (HPLC)

HPLC was used to quantify sugars and aliphatic organic acids in AL_AP_, DALs (after acid precipitation and filtration), and in all biological supernatants according to the NREL Laboratory Analytical Procedure (Sluiter et al., 2008). All samples were homogenized and filtered through a 0.22 µm Millex syringe filter (13 mm diameter), and quantification was performed by external calibration. An Agilent 1,260 Infinity high-performance liquid chromatograph equipped with a refractive index detector (35 °C) was employed for the analysis. Chromatographic separation was achieved using an Aminex HPX-87H analytical column (300 mm × 7.8 mm) with a 30 mm × 4.6 mm pre-column, maintained at 35 °C, with a flow rate of 0.6 mL min^-1^ and 5 mM sulfuric acid as the isocratic eluent.

#### 2.5.3 Carbohydrate quantification

The carbohydrate content of AL was determined after acid hydrolysis followed by HPLC analysis, according to Rocha et al. (2015). AL was subjected to acid hydrolysis (pH adjusted to 2 with 98% sulfuric acid, 121 °C for 30 min), and the resulting hydrolysate was analyzed by HPLC as described in Section 2.5.2. Appropriate correction fractions were applied to degradation products of glucose and xylose, as described in the equations below.

The glucan content (%) was calculated using Equation 3:

$$\text{Glucan}=\frac{{0.90\;x\;m_{\text{glucose}}+0.95\;x\;m_{\text{cellobiose}}+1.286\;x\;m_{5‐\text{hydroxymethylfurfural}}+3.523\;x\;m}_{\text{formic}\;\text{acid}\;}}{m_{\text{AL}}}\times 100$$ (3)

Hemicellulose content was calculated using Equation 4:

$$Hemicellulose=\frac{{0.88\;x\;m_{xylose}+0.88\;x\;m_{arabinose}+0.717\;x\;m_{acetic\;acid\;}+1.375\;x\;m}_{furfural\;}}{m_{AL}}\times 100$$ (4)

#### 2.5.4 Gas chromatography–mass spectrometry (GC-MS)

For the GC-MS analysis of aromatic monomers in AL, DALs (after acid precipitation and filtration), and all biological supernatants, a sample preparation protocol based on the method described by Martim et al. (2024) was used. Adaptations in the derivatization protocol, aimed at avoiding the use of the toxic pyridine, were compared in terms of analytical performance, including recovery, precision, and linearity, while keeping the extraction procedure identical. Specific calibration curves were constructed for each derivatization approach under the same analytical conditions. Recovery rates were close to 100% for both protocols.

##### 2.5.4.1 AL, DALs, and aerobic assay supernatants

Samples (400 µL) were acidified with 72% H_2_SO_4_ to pH 1–2, spiked with 34 µL of 3,5-dimethylphenol (1,000 μg mL^-1^ in ethyl acetate, internal standard), and extracted twice with ethyl acetate (1:1, v/v). The combined organic phases were dried with ∼50 mg MgSO_4_, and 400 µL of the extract was derivatized with 100 µL BSTFA and 100 µL pyridine at 40 °C for 15 min before GC analysis (Qi et al., 2019). Analyses were performed on a Shimadzu GC-2010 gas chromatograph coupled to a GCMS-QP2010 Ultra mass spectrometer, equipped with an Agilent DB-35M capillary column (30 m × 0.25 mm × 0.25 µm). Injector temperature was 250 °C, helium was used as carrier gas at 1.5 mL min^-1^, injection volume was 2 µL in split mode (1:5), and the oven program was set from 50 °C to 320 °C. The GC/MS interface and ion source were maintained at 280 °C and 250 °C, respectively, with EI at 70 eV. Compounds were identified as trimethylsilyl derivatives by retention times (±0.1 min relative to standards) and mass spectra and quantified using calibration curves (2.5–165 μg mL^-1^) based on analyte/internal standard peak area ratios. Calibration included 30 analytical standards (≥98% purity) from Sigma-Aldrich, Alfa Aesar, Dinâmica, Synth, and Geel (Belgium).

##### 2.5.4.2 Anaerobic assay supernatants

Samples (400 µL) were acidified with 72% H_2_SO_4_ to pH 1–2, spiked with 30 µL of 3,5-dimethylphenol (1,000 μg mL^-1^, internal standard), and extracted twice with ethyl acetate (400 μL and 370 µL). The combined organic phases were dried with ∼150 mg MgSO_4_, and 400 µL of the dried extract was derivatized with 100 µL BSTFA +0.5% TMSI at 60 °C for 40 min. Analyses were conducted on an Agilent 8890 GC coupled to a 7010C Triple Quadrupole mass spectrometer, using a DB-35M column (60 m × 0.25 mm × 0.25 µm). Injector temperature was 250 °C, helium was used as carrier gas at 1.0 mL min^-1^, injection volume was 1 µL in split mode (1:30). Calibration curves were constructed with 30 standards (≥98% purity), including phenol, guaiacol, catechol, vanillin, syringaldehyde, ferulic acid, and 4-coumaric acid.

##### 2.5.4.3 Biogas analysis

Methane (CH_4_) and carbon dioxide (CO_2_) were quantified using an Agilent Micro GC 990 equipped with molecular sieve and PPU modules. Column temperature was 80 °C, injector temperature 100 °C, and injection volume 1 mL, with argon as the carrier gas. Operating pressures were 200 kPa (molecular sieve) and 150 kPa (PPU). The system was operated using the manufacturer’s factory-tuned method for biogas analysis. According to the manufacturer’s specifications (Agilent Technologies), the system provides high analytical reliability, with area repeatability (relative standard deviation, RSD) of 0.033% and 0.070% for CH_4_ and CO_2_, respectively. These performance characteristics are suitable for the concentration ranges measured in the biogas samples of this study.

#### 2.5.5 Molar Mass Distribution estimated by SEC

Representative liquor samples were analyzed for molar mass distribution by size exclusion chromatography (SEC) using a handmade Superdex 30 Prep Grade GE column (65 cm × 1.6 cm), connected to an ÄKTA automated system equipped with a UV detector (280 nm), which allow the detection of aromatic compounds. The eluent was 0.1 M NaOH, with a flow rate of 1 mL min^-1^ at room temperature and an injection volume of 100 µL. All samples were homogenized and filtered through a 0.22 µm Millex syringe filter (13 mm diameter) prior injection.

The total column volume (V_t_) was approximately 127 mL, and the void volume (V_0_), determined using blue dextran, was approximately 48 mL. A set of standards with known molecular weights—including phenol (94 Da, Sigma-Aldrich), tannic acid (1701 Da, Sigma-Aldrich), and polystyrene sulfonate sodium salts (PSS; 246, 3,400, 6,000, and 10,000 Da, Agilent)—was used to correlate elution volume with molar mass, as elution volume increases with decreasing molecular weight in SEC. Chromatographic profiles were processed and plotted using Origin 9.0 software.

The elution volumes of the standards were as follows: phenol (94 Da; 94 mL), 4-coumaric acid (164 Da; 83 mL), tannic acid (1701 Da; 69 mL), and polystyrene sulfonate sodium salts (PSS, Agilent): 246 Da (81 mL), 3,400 Da (51 mL), 6,000 Da (49 mL), and 10,000 Da (48 mL). The chromatograms of the standards are provided in Supplementary Figure S1.

### 2.6 Statistical analysis

The effects of temperature, reaction time, and O_2_ pressure on monomer concentration, lag phase, and maximum specific growth rate (µmax) were evaluated by analysis of variance (ANOVA) of the 2^3^ full factorial design using the Protimiza Experimental Design software (Rodrigues and Iemma, 2014). Pairwise comparisons of growth parameters were performed using unpaired Welch’s t-tests, which do not assume equal variances between groups.

## 3 Results

### 3.1 Progressive shift from oligomeric to monomeric profiles upon thermochemical depolymerization

**FIGURE 2:**
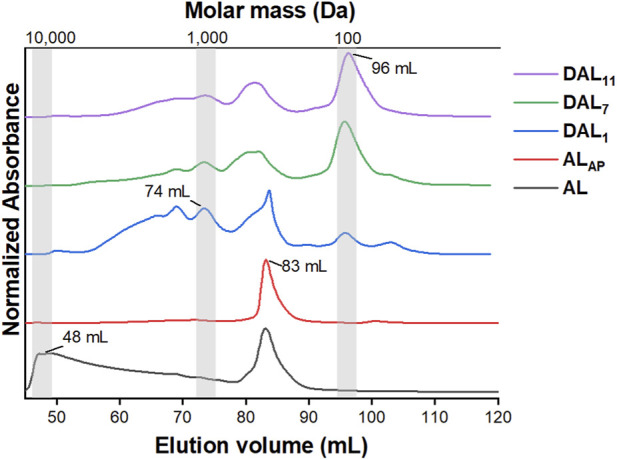
SEC chromatograms of AL, AL_AP,_ DAL_1_, DAL_7_, and DAL_11_ show the shift in molar mass distribution toward monomeric compounds with increasing reaction severity. Elution volume is inversely proportional to molar mass, with high-molecular-weight compounds eluting earlier (∼48 mL) and monomeric species later (≥83 mL). The profiles illustrate how depolymerization progressively reduces lignin molecular weight. n = 1.

The composition of AL, AL_AP_, and DAL samples were characterized using UV–vis spectroscopy, SEC, GC–MS, and HPLC (Table 2; Figure 2). According to UV–vis data, AL_AP_ presented approximately 4-fold lower concentration of total soluble aromatics relative to AL (19,000 μg mL^-1^), indicating that AL was predominantly composed of lignin fragments susceptible to acid precipitation. In agreement with the UV–vis data, SEC analysis showed that acid precipitation selectively removed macromolecular lignin from AL. AL displayed a broad molecular mass distribution, whereas AL_AP_ exhibited a well-defined low-molecular-weight peak consistent with the retention volume of the 4-coumaric acid standard (Figure 2; Supplementary Figure S1). Across the depolymerization series, SEC profiles revealed a progressive shift from oligomer-dominated to monomer-dominated profiles with increasing severity (Figure 2). According to statistical analyses, temperature was indicated as the dominant factor driving structural breakdown (Supplementary Figure S2A; Supplementary Table S1).

**TABLE 2:** Concentration of total soluble aromatics (UV–vis), monomeric aromatics (GC–MS), organic acids, and sugars (HPLC) in AL_AP_ and DALs samples. Shades of gray represent reaction temperatures ranging from 180 °C = light gray, 240 °C = medium gray, and 300 °C = dark gray. N = 1.

Concentration (µg mL^−1^)	AL_AP_	DAL_1_	DAL_2_	DAL_3_	DAL_4_	DAL_5_	DAL_6_	DAL_7_	DAL_8_	DAL_9_	DAL_10_	DAL_11_
Total aromatics (UV-vis)	5,467	1,500	1,200	1,300	1,600	1900	1800	1900	1700	2000	1,500	2000
Phenol	–	27	19	36	34	214	205	179	402	383	339	434
4-Methylphenol	–	–	–	–	–	–	–	–	11	10	11	14
4-Ethylphenol	–	–	–	–	–	8	8	7	24	23	28	28
4-Hydroxyacetophenone	–	–	–	–	15	16	–	–	17	16	16	17
4-Hydroxybenzaldehyde	28	45	32	38	46	42	38	35	38	32	22	28
4-Hydroxybenzoic acid	9	9	8	9	9	–	–	–	–	–	–	–
4-Coumaric acid	600	16	14	15	15	–	–	–	–	–	–	–
Guaiacol	–	26	16	34	30	202	184	161	341	317	195	236
4-Methylguaiacol	–	–	–	–	–	6	–	–	26	20	17	24
4-Ethylguaiacol	–	–	–	–	–	9	10	9	25	22	20	22
Acetovanillone	–	15	14	15	16	20	20	18	22	20	15	16
Vanillin	17	27	21	26	31	34	32	29	31	24	16	18
Vanillic acid	12	12	11	11	11	–	–	–	–	–	–	–
Ferulic acid	60	13	12	12	12	–	–	–	–	–	–	–
Syringol	–	–	16	35	39	202	179	161	211	191	89	83
4-Methylsyringol	–	–	–	–	–	6	6	6	29	22	14	16
Acetosyringone	–	21	17	26	24	54	57	50	42	32	16	18
Syringaldehyde	–	19	15	17	26	28	25	22	19	15	11	11
Catechol	–	–	–	–	–	11	11	11	110	87	159	244
4-Methylcatechol	3	3	3	4	3	4	4	4	26	18	34	71
3-Methoxycatechol	–	–	–	–	–	12	8	9	42	25	30	69
Acetic acid	3,106	3,058	2,331	2,749	3,165	3,212	2,993	3,039	2,347	2,859	2000	3,183
Formic acid	177	390	303	388	459	570	551	604	253	396	159	167
Glycolic acid	66	158	117	165	244	377	320	340	395	488	401	529
Lactic acid	70	297	240	340	369	893	855	913	994	1,304	1,156	1,400
Xylose	–	64	58	72	14	101	151	150	94	93	33	49
Arabinose	–	22	53	75	9	25	37	35	33	41	19	27
Glucose	–	12	20	14	–	–	–	–	15	–	–	–
∑ aliphatic acids	3,419	3,902	2,991	3,642	4,237	5,052	4,719	4,896	3,989	5,047	3,717	5,278
∑ sugars	–	97	131	161	23	126	188	184	143	134	52	75
∑ H-type monomers	637	98	73	97	118	281	251	221	493	463	415	522
∑ G-type monomers	89	92	74	98	101	272	245	218	445	404	264	317
∑ S-type monomers	–	40	48	79	89	289	268	239	300	260	129	127
∑ catechol-type monomers	3	3	3	4	3	27	23	24	178	130	223	384
∑ aromatic monomers	729	234	197	277	311	869	787	702	1,416	1,258	1,031	1,350

Reaction conditions: DAL_1_ (180 °C, 30 min, 0 bar O_2_), DAL_2_ (180 °C, 30 min, 4 bar O_2_), DAL_3_ (180 °C, 90 min, 0 bar O_2_), DAL_4_ (180 °C, 90 min, 4 bar O_2_), DAL_5_–DAL_7_ (240 °C, 60 min, 2 bar O_2_), DAL_8_ (300 °C, 30 min, 0 bar O_2_), DAL_9_ (300 °C, 30 min, 4 bar O_2_), DAL_10_ (300 °C, 90 min, 0 bar O_2_), and DAL_11_ (300 °C, 90 min, 4 bar O_2_).

To determine which specific compounds underlie the molecular mass shifts observed by SEC, aromatic monomers were quantified by GC–MS (Table 2). In AL_AP_, 4-coumaric acid was the dominant monomer, approximately 10-fold more abundant than ferulic acid, the second most abundant species. At 180 °C (DAL1–DAL4), 4-coumaric and ferulic acid concentrations dropped by approximately 40-fold and 5-fold relative to AL_AP_, respectively. Low concentrations of additional aromatic compounds—including phenol, guaiacol, and syringyl-type compounds—appeared, consistent with side-chain cleavage and the formation of dealkylated aromatic compounds.

At 240 °C (DAL5–DAL7), hydroxycinnamic and hydroxybenzoic acids were no longer detected. In contrast, phenol, guaiacol, and syringol concentrations increased roughly 6- to 10-fold relative to 180 °C. New compounds also emerged, including catechol and 3-methoxycatechol. Overall, aromatic monomer concentrations approximately tripled compared to 180 °C, suggesting enhanced lignin chain cleavage.

At 300 °C (DAL8–DAL11), aromatic monomer concentrations increased by an additional ∼1.5-fold relative to 240 °C. Phenol, guaiacol, catechol, and their alkyl-substituted variants were the most enriched compounds, consistent with intensified depolymerization and defunctionalization. Notably, catechol-type monomers, absent at 180 °C, increased by up to 15-fold between 240 °C and 300 °C, highlighting the pronounced effect of temperature on demethylation reactions.

Beyond aromatic compounds, HPLC analysis revealed that depolymerization also altered the aliphatic acid and sugar composition of the liquors. AL_AP_ contained high levels of acetic acid, consistent with hemicellulose deacetylation under mild alkaline conditions. No free sugars were detected in AL_AP_; however, after acid hydrolysis, residual glucan and xylan were quantified (Table 2).

Upon depolymerization, acetic acid levels remained relatively stable across all conditions, whereas other aliphatic acids increased markedly with severity. Lactic acid showed the most pronounced trend, increasing up to 20-fold at 300 °C relative to AL_AP_. Glycolic acid followed a similar pattern, rising up to 8-fold. Formic acid increased approximately 3-fold at 180 °C–240 °C but declined at 300 °C, suggesting its thermal degradation at higher temperatures. The formation of glycolic and lactic acids was particularly favored at 300 °C for 90 min under O_2_. Free sugars (xylose, arabinose, glucose) were detected in all DALs, indicating hydrolysis of residual carbohydrates during depolymerization. Overall, increasing severity resulted in both enrichment in aromatic monomers and a progressive increase in aliphatic organic acids.

### 3.2 Aerobic bioconversion: depolymerization of alkaline lignin improves growth of *Pseudomonas putida* KT2440

**FIGURE 3:**
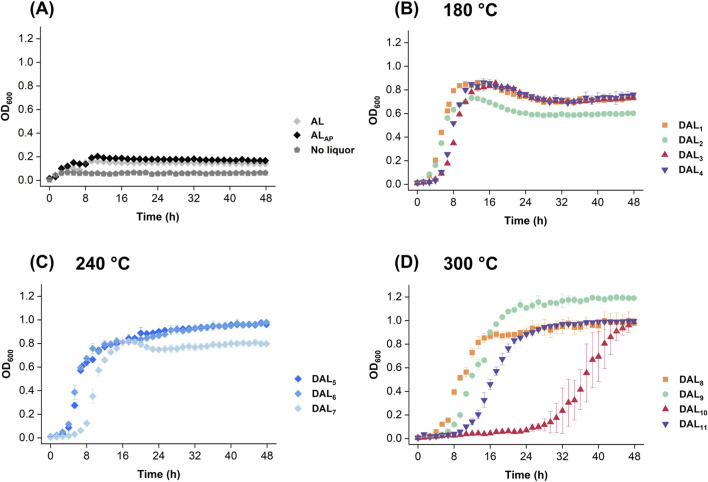
Hydrothermal treatment of alkaline liquors enhances growth of *Pseudomonas putida* KT2440. KT2440 was grown on minimal medium supplemented with lignin-rich liquors (AL, AL_AP_ and DALs) at 1 g L^-1^ of aromatic compounds (based on UV-vis estimation). **(A)** KT2440 cultures in minimal medium with AL, AL_AP_ and without supplementation of liquors are represented by the curves in light grey, black and dark grey, respectively. For DALs, the growth curves are grouped according to the temperature used for the AL depolymerization: **(B)** 180 °C, **(C)** 240 °C and **(D)** 300 °C. The graphs show mean values and standard deviations from three biological replicates.

To assess the impact of lignin depolymerization on the aerobic bioconversion of alkaline liquor, *P. putida* KT2440 (herein referred to as KT2440) was cultured in minimal medium supplemented with AL, AL_AP_ and with DALs obtained through distinct thermal treatments. AL, AL_AP_, and all DALs supported KT2440 growth. However, in DALs, OD_600_ values ranged from 0.7 to 1.2, four times higher than those observed with the liquors AL and AL_AP_ (Figure 3; Table 3).

**TABLE 3:** Growth parameters of KT2440 vary according to liquor treatment conditions.

Liquor	Depolymerization conditions	µ_max_ (h^-1^)	Lag time (h)
AL	--	0.37 ± 0.03	6.46 ± 0.19
AL_AP_	--	0.27 ± 0.04	6.36 ± 0.74
DAL_1_	180 °C, 30 min, 0 bar O_2_	0.69 ± 0.02	2.90 ± 0.05
DAL_2_	180 °C, 30 min, 4 bar O_2_	0.65 ± 0.03	3.23 ± 0.15
DAL_3_	180 °C, 90 min, 0 bar O_2_	0.55 ± 0.04	5.37 ± 0.19
DAL_4_	180 °C, 90 min, 4 bar O_2_	0.67 ± 0.03	5.09 ± 0.03
DAL_5_	240 °C, 60 min, 2 bar O_2_	0.79 ± 0.07	3.96 ± 0.09
DAL_6_	240 °C, 60 min, 2 bar O_2_	0.89 ± 0.08	3.76 ± 0.20
DAL_7_	240 °C, 60 min, 2 bar O_2_	0.77 ± 0.03	7.53 ± 0.19
DAL_8_	300 °C, 30 min, 0 bar O_2_	0.82 ± 0.09	5.86 ± 0.26
DAL_9_	300 °C, 30 min, 4 bar O_2_	0.47 ± 0.01	7.59 ± 0.05
DAL_10_	300 °C, 90 min, 0 bar O_2_	0.24 ± 0.07	28.29 ± 4.52
DAL_11_	300 °C, 90 min, 4 bar O_2_	0.33 ± 0.03	10.60 ± 0.73

Maximum specific growth rates (µ_max_) and lag times are shown for cultures grown in minimal medium supplemented with AL, AL_AP_ and DALs. Values represent the mean ± standard deviation from three biological replicates.

Among the depolymerization parameters, temperature had the most significant impact on KT2440 growth (Supplementary Figure S2B; Supplementary Table S2). Pairwise comparisons across temperature levels (DAL_1_ vs. DAL_8_, DAL_2_ vs. DAL_9_, DAL_3_ vs. DAL_10_, DAL_4_ vs. DAL_11)_ showed that cultures grown with liquors depolymerized at 300 °C exhibited significantly longer lag phases than those at 180 °C (P ≤ 0.013 for all pairs, unpaired Welch’s t-test), with average values of 13 h and 4 h, respectively (Figure 3; Table 3). At 300 °C, temperature alone did not substantially reduce µmax when combined with a short reaction time and no O_2_ supplementation (DAL_8_: 0.82 ± 0.09 h^-1^, comparable to 240 °C conditions). However, the addition of a second severity factor significantly decreased µmax (relative to DAL_8_): either extended reaction time (DAL_10:_ 0.24 ± 0.07 h^-1^, P = 0.001) or O_2_ supplementation (DAL_9_: 0.47 ± 0.01 h^-1^, P = 0.020), as well as their combination (DAL_1_1: 0.33 ± 0.03 h^-1^, P = 0.006), suggesting a synergistic effect among depolymerization parameters on substrate toxicity (Table 3).

The combined effect of temperature and reaction time also influenced KT2440 growth (Supplementary Figure S2B). Comparisons between liquors representative of different reaction times (DAL1 vs. DAL3, DAL2 vs. DAL4, DAL8 vs. DAL10, and DAL9 vs. DAL11) generally showed significantly longer lag phases (P ≤ 0.019) and lower µmax values for longer reactions, consistent with increased substrate toxicity (Table 3). Regarding oxygen availability, its effect was temperature-dependent. At 180 °C, O_2_ supplementation did not significantly affect lag phase or µmax (P > 0.05). At 300 °C, however, O_2_ supplementation in DAL11 significantly reduced the lag phase by approximately 63% compared with DAL10 (P = 0.019), although µmax was not significantly improved (P = 0.143), suggesting that O_2_ may have mitigated the initial toxicity barrier without alleviating the overall growth limitation imposed by the substrate composition (Figure 3; Table 3).

**FIGURE 4:**
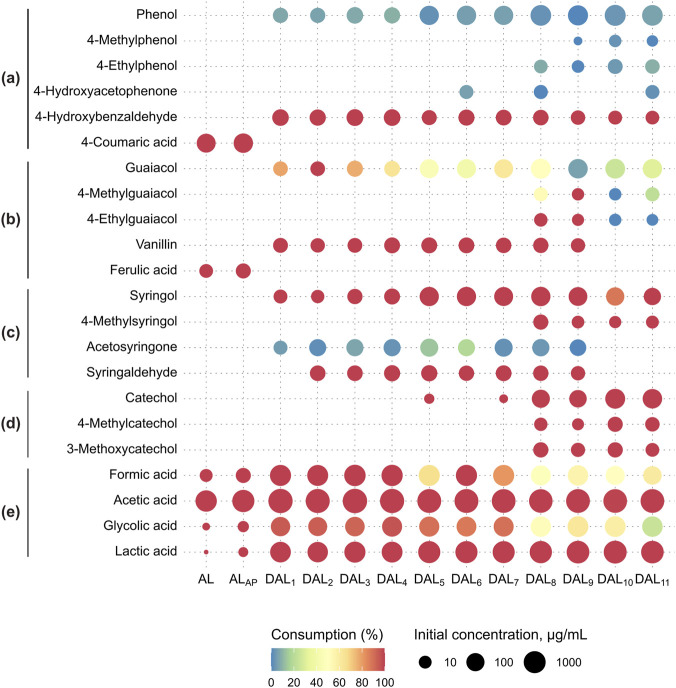
Consumption of aromatic compounds and organic acids by *Pseudomonas putida* KT2440 after 48 h cultivation in alkaline liquor (AL and AL_AP_) and depolymerized liquors (DAL_1_–DAL_11_). Each circle represents a compound detected at the start of cultivation (t = 0 h), with size proportional to its initial concentration (log scale). Circle color indicates the percentage consumed after 48 h: blue (low consumption) to red (high consumption). Absence of a circle means the compound was not detected at t = 0 h. Mean values and standard deviations (n = 3) are shown in Supplementary Tables S3–S6. The compounds were categorized in: **(a)** H-type monomers, **(b)** G-type monomers, **(c)** S-type monomers, **(d)** catechol-type monomers, and **(e)** aliphatic organic acids, as described in Supplementary Figure S3. Liquor conditions: DAL_1_ (180 °C, 30 min, 0 bar O_2_), DAL_2_ (180 °C, 30 min, 4 bar O_2_), DAL_3_ (180 °C, 90 min, 0 bar O_2_), DAL_4_ (180 °C, 90 min, 4 bar O_2_), DAL_5_–DAL_7_ (240 °C, 60 min, 2 bar O_2_), DAL_8_ (300 °C, 30 min, 0 bar O_2_), DAL_9_ (300 °C, 30 min, 4 bar O_2_), DAL_10_ (300 °C, 90 min, 0 bar O_2_), DAL_11_ (300 °C, 90 min, 4 bar O_2_).

The untreated alkaline liquor (AL and AL_AP_) primarily contained acetic, 4-coumaric, formic and ferulic acids, which served as carbon sources for KT2440 (Figure 4; Supplementary Table S3). In contrast, the depolymerized liquors (DALs) contained a broader range of detectable aromatic monomers and a higher concentration of non-aromatic compounds, such as glycolic and lactic acids (Supplementary Tables S4-S6). Most of these compounds were fully consumed by KT2440 (Figure 4; Supplementary Tables S4-S6), correlating with the enhanced bacterial growth observed in media containing DALs (Figure 3).

Among H-type monomers, KT2440 was incapable of effectively metabolizing phenol and its analogs containing alkyl or ketone side chains, whereas it completely converted those bearing aldehyde or propenoic acid side chains (Figure 4; Supplementary Figure S3). For G-type monomers, conversion of guaiacol and alkyl-substitutedte compounds varied according to the liquor composition and its initial concentration in the culture medium; the presence of aldehyde or propenoic acid side chains favored bioconversion. Among S-type monomers, all detected compounds were fully metabolized, except for acetosyringone, suggesting that the ketone group inhibits its bioconversion by KT2440 (Figure 4; Supplementary Figure S3).

In addition to aromatic compounds, all liquors contained aliphatic acids, predominantly acetic acid. Acetic and lactic acids were entirely consumed after 48 h of culturing (Figure 4). Formic acid was completely consumed in DAL_1_ to DAL_4_ liquors but only partially consumed in DAL8–DAL_11_, which were subjected to higher reaction temperatures (Figure 4; Supplementary Tables S4, S6). A similar pattern was observed for glycolic acid. Notably, the incomplete assimilation of formic and glycolic acids coincided with the liquors exhibiting the highest diversity of aromatic compounds, suggesting that the increased chemical complexity of these substrates may have negatively affected the catabolism of these aliphatic acids.

### 3.3 Anaerobic bioconversion: moderate lignin depolymerization enhances methane yield

Anaerobic bioconversion assays were conducted using four types of alkaline liquors: a non-depolymerized liquor (AL) and three different depolymerized liquors (DAL_1_, DAL_5_, and DAL_11_). DAL_1_ was produced under the mildest conditions (180 °C, 30 min, 0 bar O_2_), DAL_5_ under intermediate conditions (240 °C, 60 min, 2 bar O_2_), and DAL_11_ under the most severe treatment (300 °C, 90 min, 4 bar O_2_). For these assays, all liquors were used directly after pH adjustment to 7.0, without acid precipitation (Figure 1). It is important to note that aerobic and anaerobic bioconversion assays followed distinct standardization approaches. For aerobic assays, the liquor concentration was adjusted to 1 g L^-1^ of total soluble aromatics, based on UV-vis data (Section 2.5), whereas the anaerobic assays were standardized based on a substrate-to-inoculum ratio of 1:2, calculated using total volatile solids (TVS).

The total solids and total volatile solids in liquors ranged from 13.5 to 22.1 g L^-1^ and from 6.8 to 13 g L^-1^, respectively (Table 4). The AL exhibited the highest values for both total and volatile solids, while all depolymerized liquors showed progressively lower concentrations. This reduction is attributed to the loss of reactive mass during the depolymerization process, either through the release of gaseous byproducts or through hydrothermal carbonization, which decreases the residual solid content in the treated liquors (Long et al., 2014). In general, more severe reaction conditions resulted in lower solid content (Table 4, Supplementary Table S7), consistent with increased lignin fragmentation and greater monomer generation (Table 2).

**TABLE 4:** Total, volatile, and fixed solids (g L^-1^) in the alkaline liquor (AL), depolymerized liquors DAL_1_ (180 °C, 30 min, 0 bar O_2_), DAL_5_ (240 °C, 60 min, 2 bar O_2_), DAL_11_ (300 °C, 90 min, 4 bar O_2_), and slaughterhouse inoculum used as the microbial source.

Concentrations (g L^-1^)	AL	DAL_1_	DAL_5_	DAL_11_	Inoculum
Total solids	22.1 ± 0.1	21.2 ± 0.4	17.5 ± 0.1	13.5 ± 0.5	54 ± 3
Total volatile solids	13 ± 3	10.0 ± 0.3	8.3 ± 0.8	6.8 ± 0.5	45.1 ± 2.4
Total fixed solids	10 ± 3	11.1 ± 0.1	9.2 ± 0.8	7 ± 1	8.8 ± 0.4

These data provide insight into the physicochemical characteristics of liquors and serve as a basis for evaluating their potential as substrates in anaerobic digestion processes. Values represent the mean ± standard deviation from three replicates.

**FIGURE 5:**
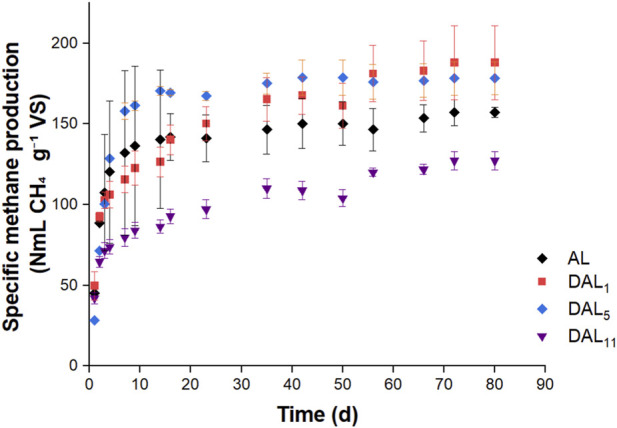
Cumulative specific methane production (NmL CH_4_ g^-1^ VS) from biochemical methane potential assays using AL (alkaline liquor) and depolymerized liquors DAL_1_ (180 °C, 30 min, 0 bar O_2_), DAL_5_ (240 °C, 60 min, 2 bar O_2_), and DAL_11_ (300 °C, 90 min, 4 bar O_2_) as sole substrates. Specific methane production represents the normalized methane volume produced per Gram of volatile solids added. Assays were conducted under standard conditions for 80 days, stabilizing after ∼62 days, with methane detectable from the first day. Data are shown as mean ± standard deviation of three biological replicates.

Among the anaerobic bioconversion systems, the mild and moderate depolymerization conditions (DAL_1_ and DAL_5_) achieved the highest cumulative specific methane yields, showing similar average values at the endpoint (188 and 178 NmL CH_4_ g^-1^ VS, respectively). These were followed by the untreated liquor (AL, 157 NmL CH_4_ g^-1^ VS) and the most severe depolymerization condition (DAL11, 127 NmL CH_4_ g^-1^ VS), which resulted in the lowest methane yield (Figure 5). Since all assays were normalized using total volatile solids as a reference, the observed differences in terms of methane yield are consistent with compositional changes associated with each thermochemical treatment.

Kinetic modelling showed a better fit for the DAL_5_ and AL systems compared to DAL_1_ and DAL_11_, likely due to the faster onset of methane production (Table 5). None of the systems showed a lag phase, indicating that the organic content required for methanogenesis was readily available. The AL system exhibited the highest maximum methane production rate (R_max_), followed by DAL_5_, DAL_1_, and DAL_11_ (Table 5). However, in terms of methane production potential, the DAL_5_ system displayed the highest methane production potential (P_max_), near 20% higher than that observed for AL (Table 5). Biogas composition varied among the systems, with the highest methane proportion observed in AL (56%), followed by DAL_5_ (37%), whereas the other DAL systems displayed values below 30% (Table 5). Together, these results show that mild to moderate depolymerization increased cumulative methane production but also shifted the biogas composition toward higher CO_2_ proportions.

**TABLE 5:** Biogas composition and kinetic parameters in the alkaline liquor (AL) and depolymerized liquors DAL1 (180 °C, 30 min, 0 bar O_2_), DAL5 (240 °C, 60 min, 2 bar O_2_), and DAL11 (300 °C, 90 min, 4 bar O_2_).

Parameter	AL	DAL_1_	DAL_5_	DAL_11_
CH_4_/CO_2_ biogas proportion (average)	56/44	25/75	37/63	20/80
P_max_ (NmL CH_4_ g^-1^ VS)	146 ± 2	159 ± 7	174 ± 2	105 ± 5
R_max_ (NmL CH_4 _g^-1^ VS d^-1^)	41 ± 3	33 ± 6	35 ± 3	26 ± 5
Lag phase λ (d)	0	0	0.1 ± 0.2	0
R^2^	0.94	0.67	0.99	0.60
RSS	789.96	7,907.54	334.54	3,625.67
RMSE	6.62	21.57	4.42	13.48

Modified Gompertz model parameters for anaerobic digestion assays. P_max_: maximum methane production potential; R_max_: maximum methane production rate. Goodness-of-fit was evaluated using the coefficient of determination (R^2^), residual sum of squares (RSS), and root mean square error (RMSE). Values are expressed as mean ± standard deviation from three biological replicates.

**FIGURE 6:**
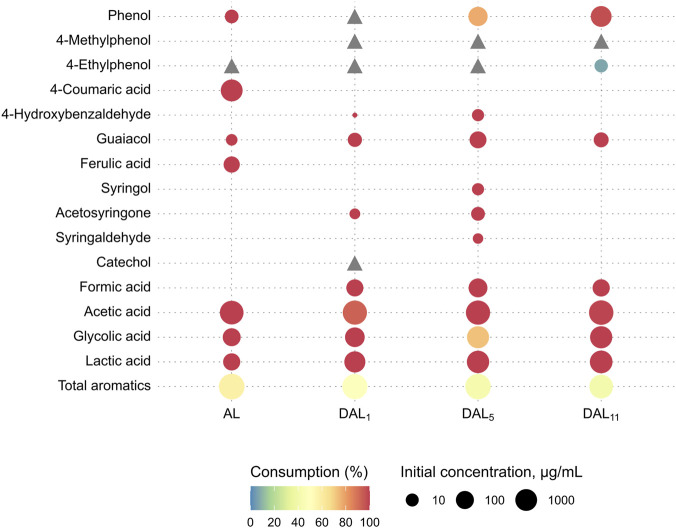
Consumption of aromatic compounds and organic acids during anaerobic reactions. Each circle represents a compound detected at the start of cultivation (*t* = 0 h), with size proportional to its concentration at *t = 0 h* (log scale). Circle color indicates the percentage of consumption of each compound at 120 days relative to *t* = 0 h. Triangles indicate compounds that were detected at higher concentration at the end of the experiment relative to *t* = 0 h. Mean values and standard deviations (n = 3) are shown in Supplementary Table S8.

The anaerobic microbial community fully or partially consumed most of the aromatic monomers and aliphatic acids detected in the tested liquors, except for 4-methylphenol and 4-ethylphenol (Figure 6; Supplementary Table S8). In some conditions, the concentrations of these compounds increased, suggesting their possible formation during the anaerobic process. For aliphatic acids, all liquors showed clear evidence of microbial consumption. Formic and lactic acids were fully depleted in the tested conditions whereas acetic and glycolic acid displayed consumption levels ranging from 73% to 100% on average (Figure 6; Supplementary Table S8).

**FIGURE 7:**
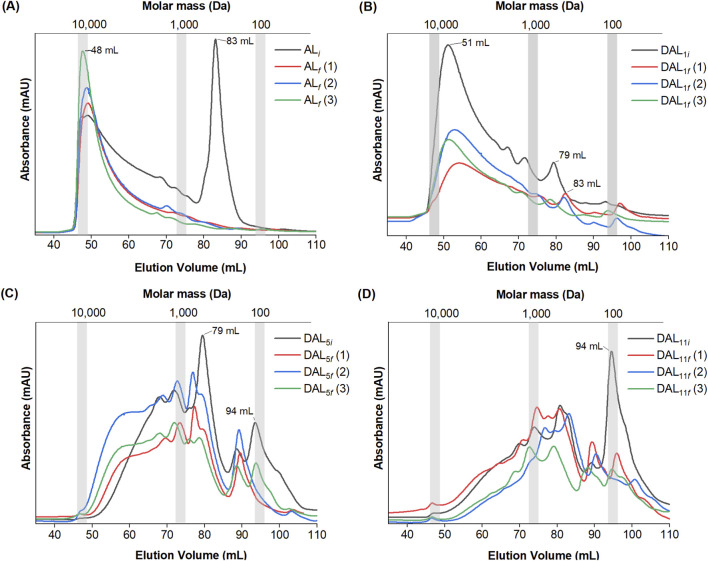
SEC profiles of liquor samples collected before and after anaerobic digestion assays. **(A)** AL (alkaline liquor from sugarcane bagasse, initial vs. final), **(B)** DAL_1_ (180 °C, 30 min, 0 bar O_2_), **(C)** DAL_5_ (240 °C, 60 min, 2 bar O_2_), and **(D)** DAL_11_ (300 °C, 90 min, 4 bar O_2_). *Initial* (i) = supernatant immediately after inoculation; *Final (f)* = supernatant after 120 days of anaerobic reaction. DAL: depolymerized alkaline liquor. Numbers (1-3) represent independent replicates. Molar mass references are highlighted in gray and were estimated based on the elution volume of standard compounds (Supplementary Figure S1).

Lignin oligomers were the most recalcitrant to the anaerobic digestion process, according to SEC analysis (Figure 7). Upon depolymerization reactions, a more heterogeneous profile of mass distribution was observed in the liquors, with a shift towards lower molecular weight fragments, including fragmentation products of monomers, such as phenol (elution volume ∼94 mL), which can be derived from 4-coumaric acid breakdown (elution volume ∼83 mL) (Figure 7; Supplementary Figure S1). Elution peaks attributed to 4-coumaric acid in the AL system and phenol in the DAL_5_ and DAL_11_ systems decreased consistently upon the anaerobic digestion, in agreement with our quantitative analysis (Figures 6, 7). However, intermediate-sized oligomers generated under moderate and high severity depolymerization conditions elicited a more heterogeneous response in the biological replicates. These results suggest that chemical modifications introduced into lignin fragments during depolymerization may impair their biodegradability by anaerobic microorganisms (Figure 7).

## 4 Discussion

The alkaline treatment of sugarcane bagasse under mild temperature conditions (<100 °C) efficiently fractionates the biomass, yielding a lignin-rich liquor (AL) and a cellulose-rich solid. However, most studies investigate the saccharification and fermentation of the cellulose-rich fraction (Freixo et al., 2023; Yaverino-Gutierrez et al., 2025)​, while the bioconvertibility of the lignin-rich liquor remains largely overlooked. This study assessed the suitability of a lignin-rich alkaline liquor, produced from sugarcane bagasse under mild temperature conditions (70 °C), for aerobic and anaerobic bioconversion. Additionally, we evaluated how thermochemical treatments applied directly to this liquor alter its chemical composition and bioconversion potential.

By varying temperature, reaction time, and oxygen pressure, we generated a series of depolymerized liquors (DAL_1_–DAL_11_) with distinct chemical profiles and degrees of lignin fragmentation. Increased hydrothermal reaction severity resulted in higher yields of monomers and short oligomers, but at the expense of degrading biocompatible monomers such as ferulic acid and 4-coumaric acid. The compositional trends observed across the depolymerization series are consistent with well-established thermochemical degradation pathways of lignin under base-catalyzed conditions. The marked decrease in hydroxycinnamic acids (4-coumaric and ferulic) at ≥180 °C is consistent with thermal degradation of the propenoic side chain, a reaction known to proceed via water-mediated decarboxylation at comparable temperatures (SriBala et al., 2021). The production of acetosyringone monomers throughout the depolymerization series is likely associated with the base-catalyzed cleavage of β-O-4 ether linkages in lignin oligomers (Mensah et al., 2022). The decline of syringol and guaiacol at the most severe conditions (DAL_10_ and DAL_11_), concomitant with the accumulation of 3-methoxycatechol and catechol, is consistent with demethylation pathways involving the hydrolysis of the O–CH_3_ bond while retaining the oxygen on the aromatic ring (Zhu et al., 2023). Overall, these concurrent pathways partially explain the chemical landscape encountered by microorganisms in subsequent bioconversion assays. A deeper understanding of the interplay between inter-unit bond cleavage and intra-monomer defunctionalization will be essential to predict and control the biological compatibility of lignin-derived streams.

Although the aerobic and anaerobic assays were performed with and without prior acid precipitation of macromolecular lignin, respectively—precluding direct comparison of overall bioconversion performance between routes—they nonetheless enabled qualitative comparison of monomer-specific recalcitrance. For instance, 4-ethylphenol and 4-methylphenol were resistant to bioconversion in both systems. Phenol is known to inhibit anaerobic digestion at concentrations near 1,400 μg mL^-1^ (Chapleur et al., 2016), likely through disruption of microbial membranes (Ko et al., 2015). However, in most of our anaerobic assays, phenol (<271 μg mL^-1^) was either partially or fully consumed, suggesting that the anaerobic consortium was able to utilize it as a substrate for methanogenesis, consistent with previous reports (Sampaio et al., 2011; Trujillo-Reyes et al., 2019; Caroca et al., 2021). This contrasts with the aerobic assays, where phenol was recalcitrant to *P. putida* KT2440 (Figures 4, 6).

The formation of small amounts of 4-methylphenol and 4-ethylphenol was detected in some anaerobic assays, which may reflect the transformation of complex aromatics into simpler phenolic structures by the microbial community (Barakat et al., 2012)​. Similar mechanisms have been reported for other compounds, such as the conversion of methylfurans into furfural by methanogenic archaea like *Methanococcus* (Balasundaram et al., 2022). Interestingly, acetosyringone, which is not metabolized by *P. putida* KT2440, was efficiently consumed in anaerobic assays, suggesting that the microbial community used in this study could be a promising source of enzymes to enable acetosyringone metabolism in engineered cell factories. However, beyond identifying these enzymes, further studies will be required to assess the compatibility of this metabolic route for applications in aerobic bacteria such as *P. putida*.

For *P. putida* growth, the best results were observed under mild to moderate depolymerization conditions, which correlate with the lower content of toxic monomers such as phenol and its alkyl-substituted variants, and catechol. Growth inhibition of *P. putida* strains was reported to phenol and catechol individually added at concentrations of 600 μg mL^-1^and 770 μg mL^-1^, respectively (Santos et al., 2004; Kohlstedt et al., 2018). At the severe depolymerization conditions, although phenol and catechol concentrations are below this levels (<300 μg mL^-1^), the observed inhibition of bacterial growth in our experiments is most likely due to the combined toxic effects of the compound mixture, as previously reported (Pienkos and Zhang, 2009; Menezes et al., 2022). To improve its tolerance and efficiency in metabolizing lignin-derived compounds generated in the reactions at 300 °C, particularly in relation to DAL_10_ and DAL_11_, metabolic engineering strategies will be essential to overcome the bottleneck of guaiacol and phenol metabolism in the KT2440 platform. In this context, heterologous expression of guaiacol *O-*demethylase and phenol hydroxylase represents promising approaches, as demonstrated in previous studies (Kohlstedt et al., 2018; Tumen-Velasquez et al., 2018; Bleem et al., 2022).

In the anaerobic digestion assays, the moderate depolymerization condition (DAL5) yielded the highest methane production, with approximately 20% increase compared to the untreated alkaline liquor. This result suggests that moderate depolymerization provides the most favorable balance between substrate accessibility and biological compatibility. However, the proportion of CO_2_ in the biogas increased across all DAL samples relative to AL (Table 5), suggesting impairment of methanogenic pathways. For this phenomenon, we hypothesize a dual inhibition mechanism: recalcitrant monomers, particularly phenol and its alkyl-substituted variants, may have selectively impaired acetoclastic methanogenesis (Olguin-Lora et al., 2003; Li et al., 2025), prompting a compensatory shift toward syntrophic acetate oxidation (SAO) coupled with hydrogenotrophic methanogenesis (Muñoz Sierra et al., 2020; Prem et al., 2021). However, in this two-step route, the CO_2_ produced as an intermediate by SAO may not be efficiently converted to CH_4_ during hydrogenotrophic methanogenesis, leading to its direct accumulation in the biogas. Furthermore, incomplete H_2_ consumption by inhibited hydrogenotrophic archaea may raise the H_2_ partial pressure, rendering SAO thermodynamically unfavorable (Hattori, 2008) and resulting in residual acetate accumulation, consistent with the acetate detected at the end of the DAL1 and DAL11 assays (Supplementary Table S8). This dual bottleneck model—inhibition of both acetoclastic and hydrogenotrophic methanogenesis—provides a coherent framework to explain the shift in biogas composition, and the progressive decrease in maximum methane production rates (Rmax) with increasing depolymerization severity. Elucidating the precise contribution of each mechanism will require future studies combining microbial community profiling with pathway-specific activity assays.

## 5 Conclusion and perspectives

In this study, we show that thermochemical depolymerization severity strongly shapes the chemical profile of a lignin-rich alkaline liquor and, consequently, its performance in aerobic and anaerobic bioconversion. Increasing severity promotes a shift from oligomeric to monomer-rich lignin fractions; however, enhanced chemical depolymerization does not linearly improve biological conversion. Instead, mild to moderate depolymerization conditions provided the most favorable balance between lignin fragmentation and biological compatibility, improving both *P. putida* KT2440 growth and methane production, whereas severe conditions generated streams containing higher amounts of recalcitrant monomers—particularly phenol and its alkyl-substituted variants—associated with microbial growth inhibition and reduced methanogenesis. Together, our results indicate that lignin depolymerization alone is insufficient to maximize bioconversion efficiency and highlight toxicity and compound-specific recalcitrance as key limiting factors. Future research should focus on integrating controlled depolymerization with metabolic engineering strategies to expand microbial bioconversion capacity, as well as on microbial community analyses, process optimization, techno-economic evaluation, and life cycle assessment to support the scalable and sustainable integration of these lignin valorization routes within biorefinery schemes.

## Data Availability

The original contributions presented in the study are included in the article/Supplementary Material, further inquiries can be directed to the corresponding authors.
