# Supplementary material for: Impact of lignin depolymerization on aerobic and anaerobic bioconversion of alkaline liquor from sugarcane bagasse
Source: Front Bioeng Biotechnol. 2026 Jul 14;14:1837262. doi: 10.3389/fbioe.2026.1837262 (PMC13407638; doi:10.3389/fbioe.2026.1837262)
Supplement: Supplementary file 1 [file DataSheet1.docx]

Supplementary Information

Impact of lignin depolymerization on aerobic and anaerobic bioconversion of alkaline liquor from sugarcane bagasse

**Fabrícia Farias de Menezes^1,†^, Fernanda Miyuki Kashiwagi^1,†^, Jessica Jacinta Silva^2^, Gustavo Rodrigues Gomes^1^, Rafaela Prata^1^, Maria Rosa de Moraes^1^, Adriano Freitas Lima^1^, Felipe Garcia da Silva^1^, Renata Piacentini Rodriguez^2^, Carlos Eduardo Driemeier^1^, George Jackson de Moraes Rocha^1*,† †^, Priscila Oliveira Giuseppe^1*,† †^**

^1^Brazilian Biorenewables National Laboratory (LNBR), Brazilian Center for Research in Energy and Materials (CNPEM), Campinas, Brazil

^2^Institute of Science and Technology (ICT), Federal University of Alfenas (UNIFAL), Poços de Caldas, Brazil

† These authors contributed equally to this work and share first authorship.

†† These authors share last authorship.

*** Correspondence:**
George Jackson de Moraes Rocha

[george.rocha@lnbr.cnpem.br](mailto:george.rocha@lnbr.cnpem.br)

Priscila Oliveira de Giuseppe

[priscila.giuseppe@lnbr.cnpem.br](mailto:priscila.giuseppe@lnbr.cnpem.br)


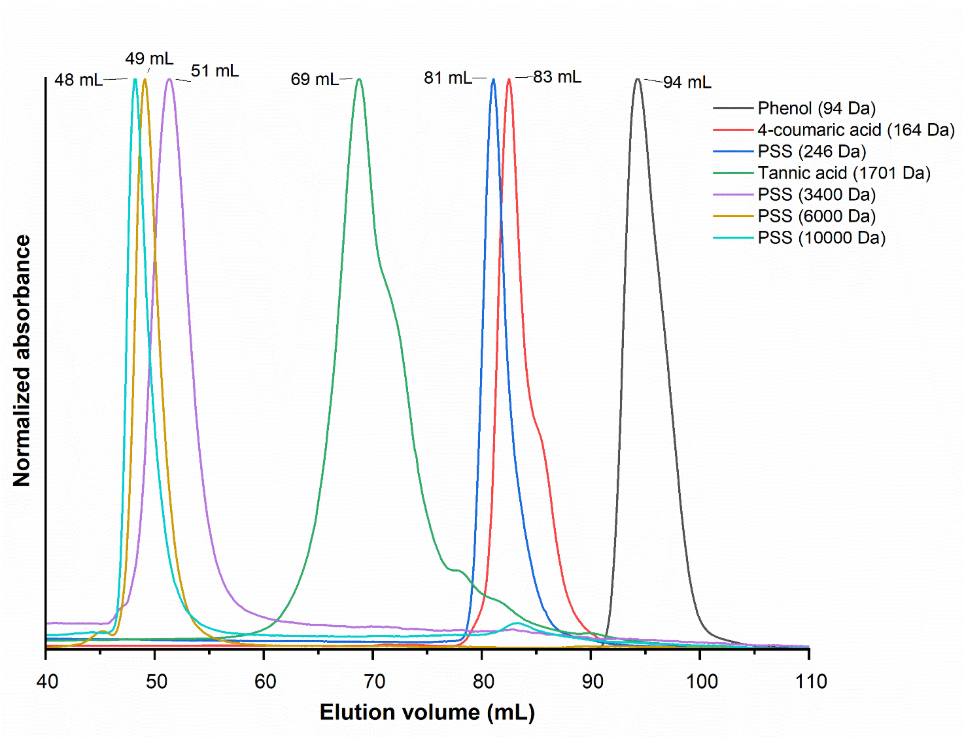


**Figure S1.** **SEC profiles of molecular mass standards.** The standards used to correlate elution volume with molecular mass were phenol (94 Da, 94 mL), tannic acid (1701 Da, 69 mL), and polystyrene sulfonate sodium salts (PSS, Agilent; 246 Da, 81 mL; 3400 Da, 51 mL; 6000 Da, 49 mL; and 10,000 Da, 48 mL).


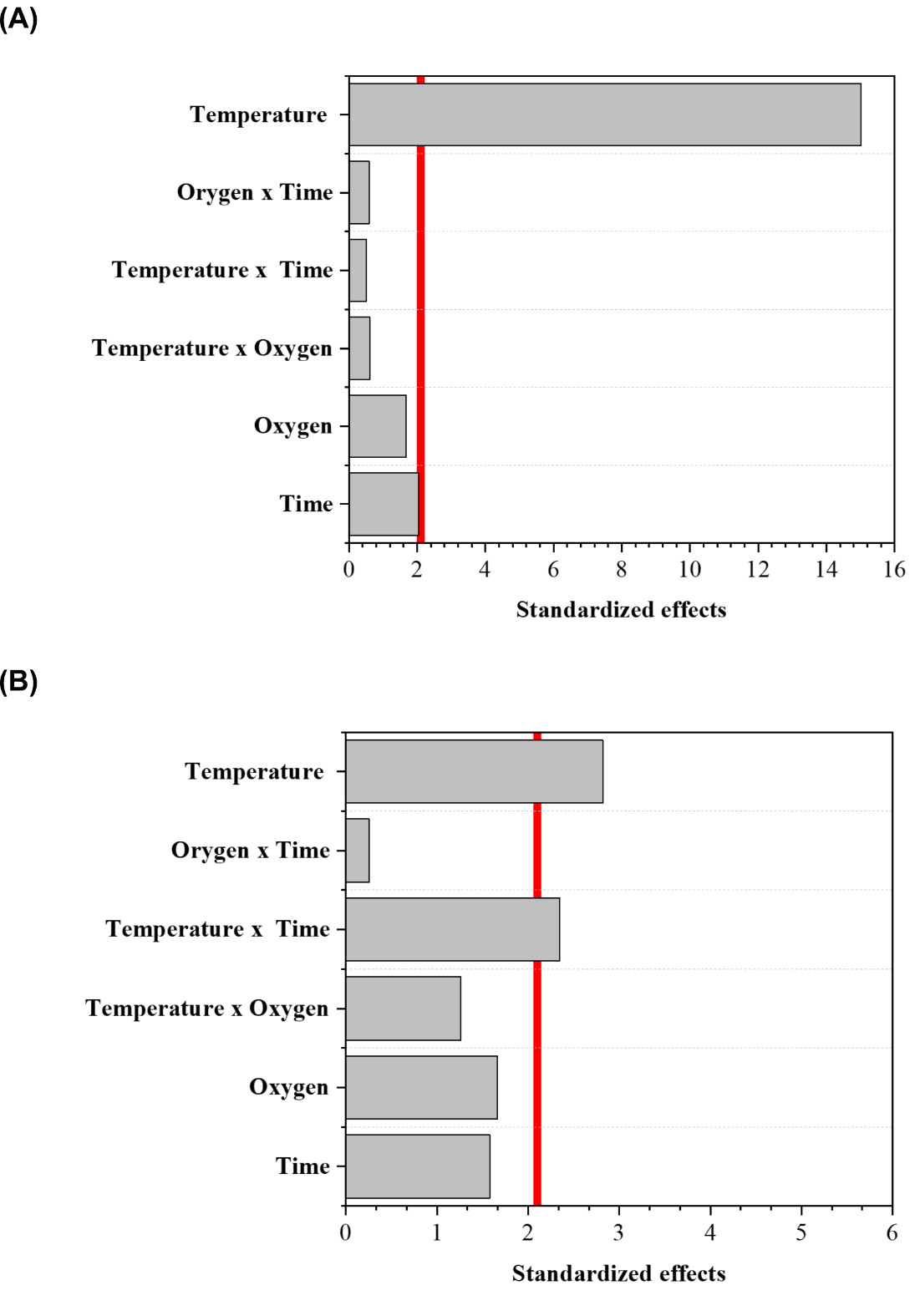


**Figure S2.** **Pareto charts showing the standardized effects of the process variables: temperature, oxygen addition, and reaction time**, **on (A) aromatic monomer concentration (µg mL⁻¹) and (B) lag time (h), obtained from a** $\boldsymbol{2}^{\boldsymbol{3}}$ **factorial experimental design with triplicates at the central point (11 runs).** Statistical significance was evaluated at a 90% confidence level ($p<0.10$). The effect estimates were calculated using PROTIMIZA software, and the Pareto charts were generated using Origin software.


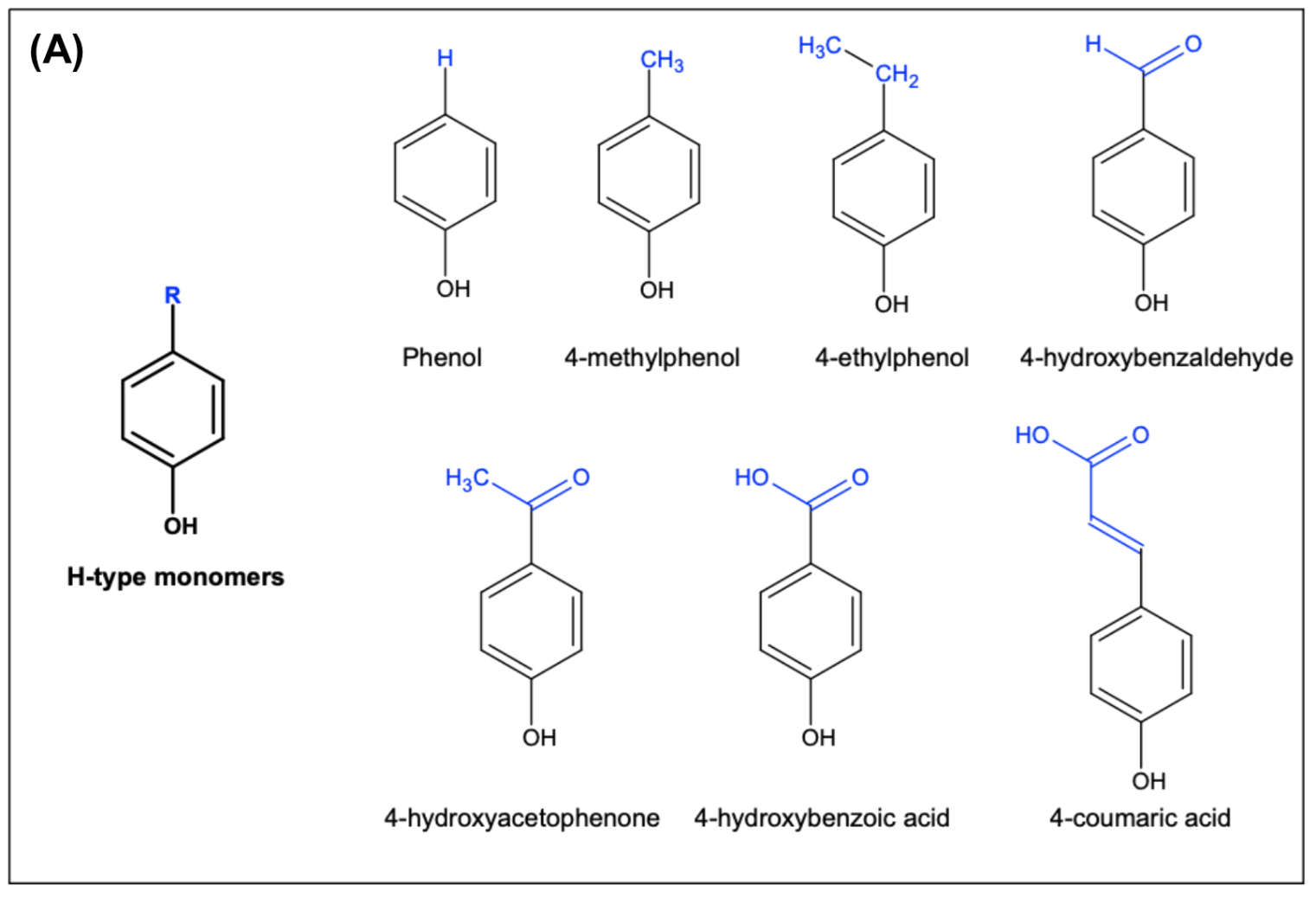

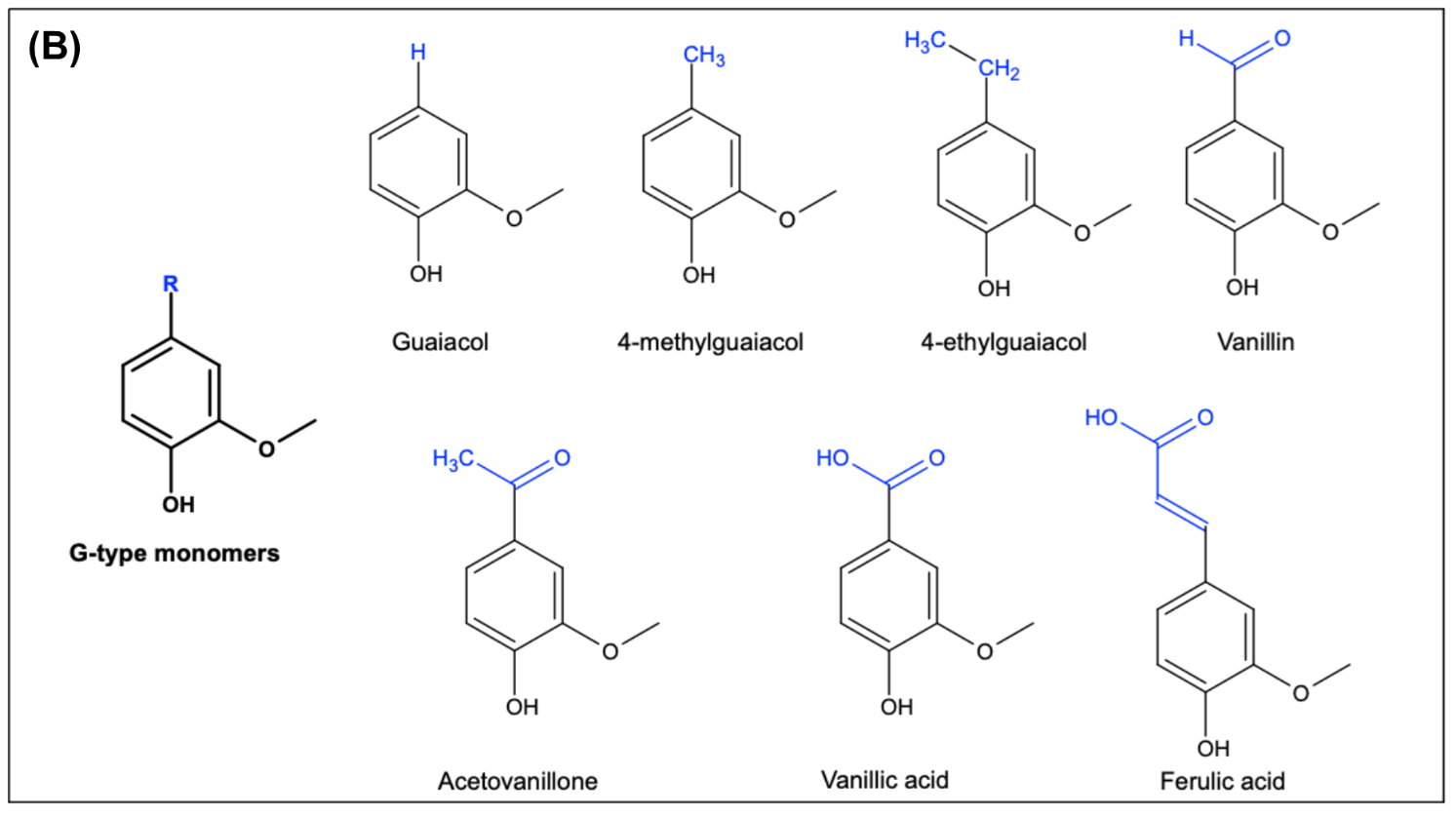


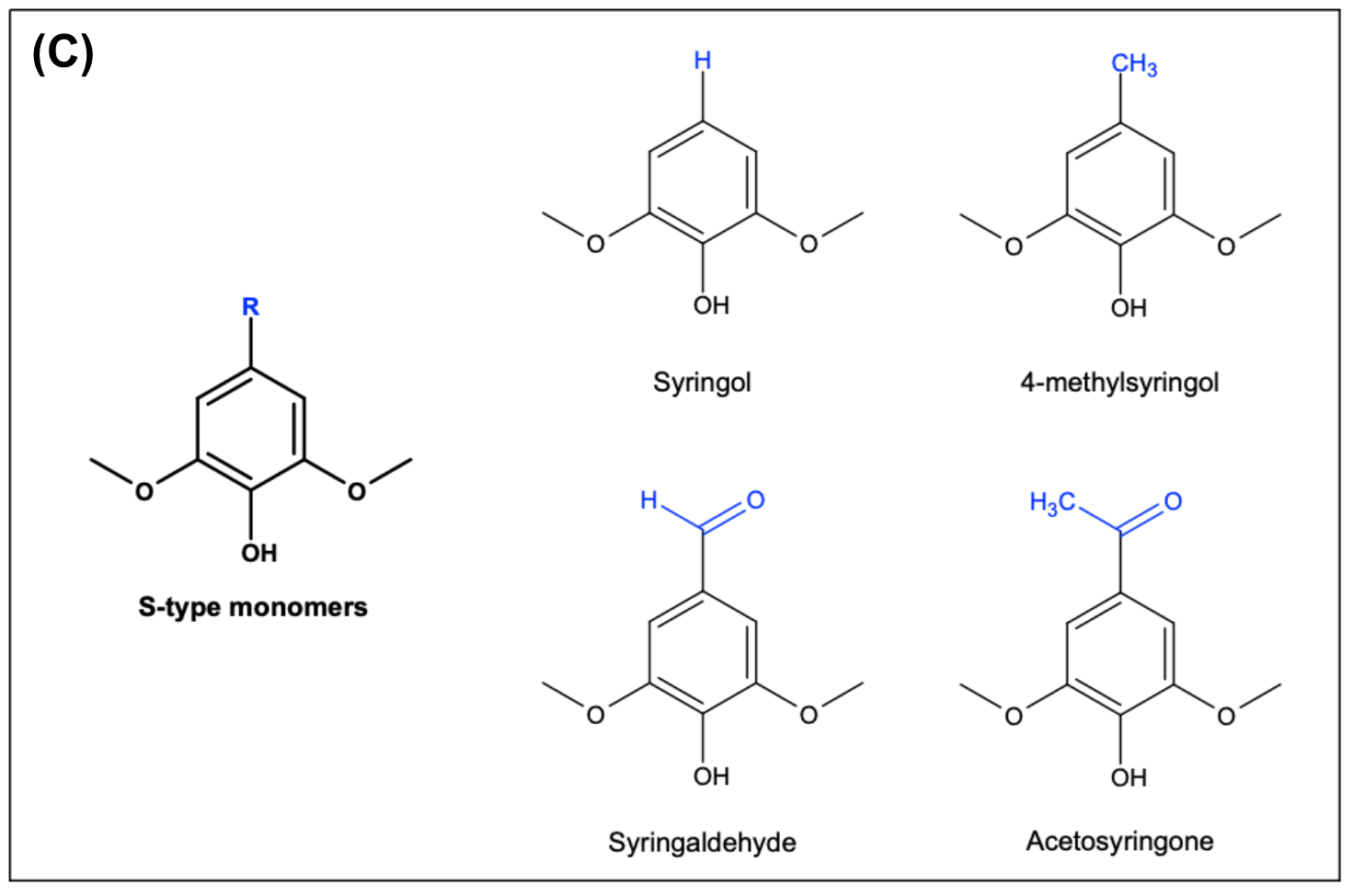


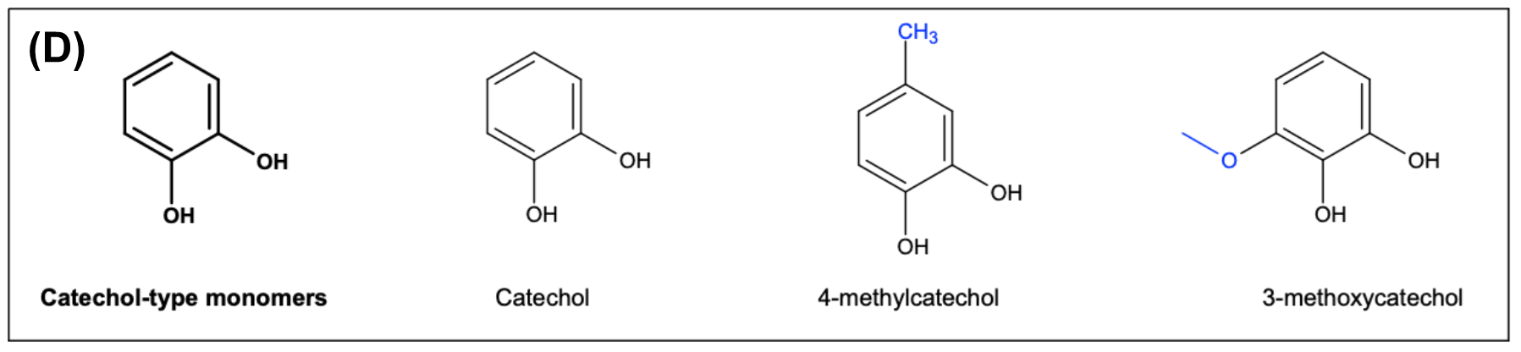


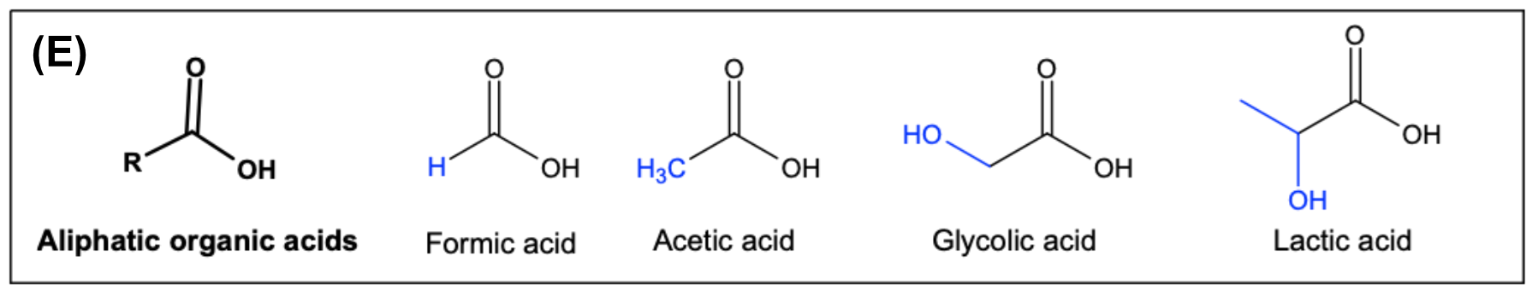


**Figure S3. Chemical structures of aromatic monomers and aliphatic compounds present in treated or untreated alkaline liquors.** Aromatic monomers were divided into four categories: (A) H-type monomers with a *p-*hydroxyphenyl backbone, (B) G-type monomers with a guaiacyl backbone, (C) S-type monomers with a syringyl backbone, and (D) catechol-type monomers. Aliphatic organic acids are shown in (E).

# Supplementary Tables

**Table S1**. **Analysis of variance (ANOVA) for the** $\boldsymbol{2}^{\boldsymbol{3}}$**factorial experimental design with triplicates at the central point (11 runs), evaluating the response of total aromatic monomer concentration (µg mL⁻¹) according to GC-MS data.** The regression model was statistically significant at a 90% confidence level ($p<0.10$) and showed a high coefficient of determination ($R^{2}=98.32\%$), with no significant lack of fit ($p>0.10$). The values estimates were calculated using the PROTIMIZA software.

| **Source of Variation** | **Sum of Squares** | **Degrees of Freedom** | **Mean Square** | **Fcalc** | **p-value** |
| --- | --- | --- | --- | --- | --- |
| Regression | 2107807.0 | 6 | 351301.2 | 38.9 | 0.00166 |
| Residuals | 36111.7 | 4 | 9027.9 |  |  |
| Lack of Fit | 22165.7 | 2 | 11082.9 | 1.6 | 0.38619 |
| Pure Error | 13946.0 | 2 | 6973.0 |  |  |
| Total | 2143918.7 | 10 |  |  |  |

**Table S2. Analysis of variance (ANOVA) for the 2^3^ factorial experimental design with triplicates at the central point (11 runs), evaluating the response of lag time (h).** The regression model was statistically significant at a 90% confidence level (𝑝 < 0.10) and showed a coefficient of determination (𝑅^2^=84.54 %) with no significant lack of fit (𝑝 > 0.10). The values estimates were calculated using the PROTIMIZA software.

| **Source of Variation** | **Sum of Squares** | **Degrees of Freedom** | **Mean Square** | **Fcalc** | **p-value** |
| --- | --- | --- | --- | --- | --- |
| Regression | 440.1 | 6 | 73.3 | 3.6 | 0.11552 |
| Residuals | 80.5 | 4 | 20.1 |  |  |
| Lack of Fit | 71.5 | 2 | 35.7 | 7.9 | 0.11185 |
| Pure Error | 9.0 | 2 | 4.5 |  |  |
| Total | 520.5 | 10 |  |  |  |

**Table S3. Concentration of aromatic compounds and organic acids measured during *P. putida* KT2440 growth.**Measurements were taken at t_i_ = 0 h and t_f_ = 48 h post inoculation in minimal medium supplemented with AL or AL_AP_. AL: Alkaline liquor; AL_AP_: Alkaline liquor after acid precipitation up to pH 2. Data are shown as mean ± SD (n=3), except for 4-coumaric acid and ferulic acid (n=1).

| **Compounds (µg mL^-1^)** | **AL** | | **AL_AP_** | | |
| --- | --- | --- | --- | --- | --- |
|  | **t*_i_*** | **t*_f_*** | **t*_i_*** | **t*_f_*** |  |
| **Phenol** | -- | -- | -- | -- |  |
| **4-Methylphenol** | -- | -- | -- | -- |  |
| **4-Ethylphenol** | -- | -- | -- | -- |  |
| **4-Hydroxyacetophenone** | -- | -- | -- | -- |  |
| **4-Hydroxybenzaldehyde** | -- | -- | -- | -- |  |
| **4-Coumaric acid** | 90.6 | -- | 120.6 | -- |  |
| **Guaiacol** | -- | -- | -- | -- |  |
| **4-Methylguaiacol** | -- | -- | -- | -- |  |
| **4-Ethylguaiacol** | -- | -- | -- | -- |  |
| **Vanillin** | -- | -- | -- | -- |  |
| **Ferulic acid** | 10.0 | -- | 6.5 | -- |  |
| **Syringol** | -- | -- | -- | -- |  |
| **4-Methylsyringol** | -- | -- | -- | -- |  |
| **Acetosyringone** | -- | -- | -- | -- |  |
| **Syringaldehyde** | -- | -- | -- | -- |  |
| **Catechol** | -- | -- | -- | -- |  |
| **4-Methylcatechol** | -- | -- | -- | -- |  |
| **3-Methoxycatechol** | -- | -- | -- | -- |  |
| **Acetic acid** | 361.2 ± 3.9 | -- | 629.3 ± 3.2 | -- |  |
| **Formic acid** | 7.6 ± 0.5 | -- | 15.8 ± 1.5 | -- |  |
| **Glycolic acid** | 2.6 ± 0.3 | -- | 4.9 ± 0.3 | -- |  |
| **Lactic acid** | 2.2 ± 0.4 | -- | 3.6 ± 0.9 | -- |  |

**Table S4. Concentration of aromatic compounds and organic acids measured during *P. putida* KT2440 growth.**Measurements were taken at t_i_ = 0 h and t_f_ = 48 h post inoculation in minimal medium supplemented with DAL_1_, DAL_2_, DAL_3_, or DAL_4_. Reaction conditions: DAL_1_ (180 °C, 30 min, 0 bar O₂), DAL_2_ (180 °C, 30 min, 4 bar O₂), DAL_3_ (180 °C, 90 min, 0 bar O₂), DAL_4_ (180 °C, 90 min, 4 bar O₂). “--”: below detection limit or not detected. Data are shown as mean ± SD of triplicates.

| **Compounds (µg mL^-1^)** | **DAL_1_** | | **DAL_2_** | | **DAL_3_** | | **DAL_4_** | |
| --- | --- | --- | --- | --- | --- | --- | --- | --- |
|  | **t*_i_*** | **t*_f_*** | **t*_i_*** | **t*_f_*** | **t*_i_*** | **t*_f_*** | **t*_i_*** | **t*_f_*** |
| **Phenol** | 17.3 ± 0.5 | 16.0 ± 0.6 | 16.6 ± 0.4 | 15.5 ± 0.4 | 29.8 ± 0.1 | 27.5 ± 0.5 | 24.7 ± 0.3 | 22.3 ± 0.5 |
| **4-Methylphenol** | -- | -- | -- | -- | -- | -- | -- | -- |
| **4-Ethylphenol** | -- | -- | -- | -- | -- | -- | -- | -- |
| **4-Hydroxyacetophenone** | -- | -- | -- | -- | -- | -- | -- | -- |
| **4-Hydroxybenzaldehyde** | 29.4 ± 0.3 | -- | 25.2 ± 1.5 | -- | 32.4 ± 2.5 | -- | 30.6 ± 0.8 | -- |
| **Guaiacol** | 14.0 ± 0.3 | 3.0 ± 2.7 | 13.5 ± 0.5 | -- | 24.1 ± 0.7 | 5.5 ± 0.5 | 21.2 ± 0.5 | 7.6 ± 1.4 |
| **4-Methylguaiacol** | -- | -- | -- | -- | -- | -- | -- | -- |
| **4-Ethylguaiacol** | -- | -- | -- | -- | -- | -- | -- | -- |
| **Vanillin** | 15.0 ± 2.1 | -- | 12 ± 11 | -- | 14 ± 12 | -- | 21.9 ± 1.2 | -- |
| **Syringol** | 10.4 ± 3.0 | -- | 8.5 ± 2.6 | -- | 17± 15 | -- | 22.4 ± 0.7 | -- |
| **4-Methylsyringol** | -- | -- | -- | -- | -- | -- | -- | -- |
| **Acetosyringone** | 11 ± 19 | 10 ± 18 | 35.8 ± 0.5 | 35.2 ± 0.2 | 38.5 ± 1.2 | 35.8 ± 1.0 | 38.6 ± 1.3 | 37.6 ± 0.8 |
| **Syringaldehyde** | -- | -- | 19.6 ± 0.5 | -- | 20.0 ± 0.6 | -- | 24.7 ± 1.0 | -- |
| **Catechol** | -- | -- | -- | -- | -- | -- | -- | -- |
| **4-Methylcatechol** | -- | -- | -- | -- | -- | -- | -- | -- |
| **3-Methoxycatechol** | -- | -- | -- | -- | -- | -- | -- | -- |
| **Acetic acid** | 2146 ± 31 | -- | 2732 ± 22 | -- | 2392.7 ± 6.8 | -- | 1518.3 ± 6.9 | -- |
| **Formic acid** | 280.8 ± 9.5 | -- | 372 ± 31 | -- | 351.4 ± 9.6 | -- | 159.0 ± 3.1 | 83.0 ± 0.2 |
| **Glycolic acid** | 93.4 ± 0.5 | 5.7 ± 0.1 | 127.6 ± 0.7 | 9.8 ± 0.1 | 175.0 ± 0.5 | 6.7 ± 0.1 | 206.4 ± 1.0 | 99.4 ± 2.0 |
| **Lactic acid** | 214.1 ± 2.7 | -- | 313.2 ± 4.9 | -- | 277.4 ± 3.8 | -- | 665.3 ± 9.5 | -- |

**Table S5. Concentration of aromatic compounds and organic acids measured during *P. putida* KT2440 growth.**Measurements were taken at t*_i_* = 0 h and t*_f_* = 48 h post inoculation in minimal medium supplemented with DAL_5_, DAL_6_, DAL_7_. Reaction conditions: DAL_5_–DAL_7_ (240 °C, 60 min, 2 bar O₂). “--”: below detection limit or not detected. Data are shown as mean ± SD of triplicates.

| **Compounds (µg mL^-1^)** | **DAL_5_** | | **DAL_6_** | | **DAL_7_** | |
| --- | --- | --- | --- | --- | --- | --- |
|  | **t*_i_*** | **t*_f_*** | **t*_i_*** | **t*_f_*** | **t*_i_*** | **t*_f_*** |
| **Phenol** | 106.9 ± 1.4 | 104.5 ± 1.6 | 116.8 ± 6.2 | 110.6 ± 8.8 | 105.8 ± 1.8 | 99.8 ± 3.7 |
| **4-Methylphenol** | -- | -- | -- | -- | -- | -- |
| **4-Ethylphenol** | -- | -- | -- | -- | -- | -- |
| **4-Hydroxyacetophenone** | -- | -- | 11 ± 0.2 | 10 ± 0.2 | -- | -- |
| **4-Hydroxybenzaldehyde** | 15 ± 13 | -- | 23.6 ± 1.0 | -- | 20.5 ± 1.2 | -- |
| **4-Coumaric acid** | -- | -- | -- | -- | -- | -- |
| **Guaiacol** | 91.8 ± 2.2 | 50.2 ± 1.5 | 100.6 ± 3.6 | 59.9 ± 0.6 | 88.6 ± 1.9 | 33.6 ± 6.6 |
| **4-Methylguaiacol** | -- | -- | -- | -- | -- | -- |
| **4-Ethylguaiacol** | -- | -- | -- | -- | -- | -- |
| **Vanillin** | 21.7 ± 0.2 | -- | 21.0 ± 0.6 | -- | 18.3 ± 3.0 | -- |
| **Ferulic acid** | -- | -- | -- | -- | -- | -- |
| **Syringol** | 104.0 ± 1.0 | -- | 105.9 ± 3.2 | -- | 91.1 ± 2.3 | -- |
| **4-Methylsyringol** | -- | -- | -- | -- | -- | -- |
| **Acetosyringone** | 58.4 ± 1.6 | 50.4 ± 4.5 | 36 ± 20 | 29 ± 20 | 56.4 ± 1.5 | 55.1 ± 1.2 |
| **Syringaldehyde** | 24.4 ± 0.3 | -- | 17.6 ± 4.6 | -- | 21.1 ± 2.2 | -- |
| **Catechol** | 3.9 ± 0.1* | -- | -- | -- | 3.0 ± 0.1 | -- |
| **4-Methylcatechol** | -- | -- | -- | -- | -- | -- |
| **3-Methoxycatechol** | -- | -- | -- | -- | -- | -- |
| **Acetic acid** | 1881 ± 85 | -- | 1677.0 ± 9.2 | -- | 237.2 ± 2.2 | -- |
| **Formic acid** | 314 ± 15 | -- | 302.2 ± 0.2 | 56 ± 22 | 15.0 ± 0.5 | -- |
| **Glycolic acid** | 148.5 ± 9.5 | 18.8 ± 0.5 | 163.6 ± 2.3 | 17.2 ± 0.5 | 5.2 ± 0.2 | -- |
| **Lactic acid** | 582 ± 24 | -- | 549.2 ± 4.3 | -- | -- | -- |

**Table S6. Concentration of aromatic compounds and organic acids measured during *P. putida* KT2440 growth.**Measurements were taken at t_i_ = 0 h and t_f_ = 48 h in minimal medium supplemented with DAL_8_, DAL_9_, DAL_10_, or DAL_11_. Reaction conditions: DAL_8_ (300 °C, 30 min, 0 bar O₂), DAL_9_ (300 °C, 30 min, 4 bar O₂), DAL_10_ (300 °C, 90 min, 0 bar O₂), DAL_11_ (300 °C, 90 min, 4 bar O₂). “--”: below detection limit or not detected. Data are shown as mean ± SD of triplicates.

| **Compounds (µg mL^-1^)** | **DAL_8_** | | **DAL_9_** | | **DAL_10_** | | **DAL_11_** | |
| --- | --- | --- | --- | --- | --- | --- | --- | --- |
|  | **t*_i_*** | **t*_f_*** | **t*_i_*** | **t*_f_*** | **t*_i_*** | **t*_f_*** | **t*_i_*** | **t*_f_*** |
| **Phenol** | 245.7 ± 1.4 | 239.6 ± 7.4 | 261.1 ± 6.0 | 237.7 ± 5.5 | 283.0 ± 3.1 | 273.5 ± 4.4 | 234 ± 19 | 219 ± 23 |
| **4-Methylphenol** | -- | -- | 3.0 ± 2.6 | 4.1 ± 0.2 | 6.8 ± 0.2 | 6.6 ± 0.2 | 5.1 ± 4.4 | 6.8 ± 0.3 |
| **4-Ethylphenol** | 9.9 ± 0.1 | 9.1 ± 0.5 | 6.9 ± 6.0 | 9.5 ± 0.3 | 15.2 ± 0.4 | 14.6 ± 0.3 | 13.1 ± 0.6 | 11.9 ± 0.4 |
| **4-Hydroxyacetophenone** | 11.2 ± 0.9 | 11.6 ± 0.3 | -- | -- | -- | -- | 10.8 ± 0.7 | 10.5 ± 0.5 |
| **4-Hydroxybenzaldehyde** | 19.3 ± 3.8 | -- | 13 ± 11 | -- | 10.5 ± 9.1 | -- | 9.8 ± 8.5 | -- |
| **Guaiacol** | 169.0 ± 2.2 | 84 ± 73 | 122 ± 105 | 114.2 ± 0.8 | 142.1 ± 2.4 | 106.8 ± 6.8 | 115.1 ± 4.7 | 79.6 ± 5.8 |
| **4-Methylguaiacol** | 10.9 ± 0.1 | 5.2 ± 4.5 | 6.5 ± 5.6 | -- | 6.6 ± 5.7 | 8.1 ± 0.4 | 11.2 ± 0.7 | 8.7 ± 0.5 |
| **4-Ethylguaiacol** | 9.1 ± 0.1 | -- | 6.1 ± 5.3 | -- | 6.5 ± 5.6 | 7.6 ± 0.3 | 5.5 ± 4.8 | 6.4 ± 0.2 |
| **Vanillin** | 17.5 ± 2.3 | -- | 12.1 ± 10.5 | -- | -- | -- | -- | -- |
| **Syringol** | 111.9 ± 0.8 | -- | 82 ± 71 | -- | 65.8 ± 0.5 | 7.8 ± 3.2 | 39.8 ± 1.1 | -- |
| **4-Methylsyringol** | 16.3 ± 3.9 | -- | 7.1 ± 6.6 | -- | 6.2 ± 5.4 | -- | 7.5 ± 0.5 | -- |
| **Acetosyringone** | 41.1 ± 1.3 | 39.7 ± 2.4 | 29 ± 25 | 42.6 ± 0.2 | -- | -- | -- | -- |
| **Syringaldehyde** | 18.0 ± 0.3 | -- | 12 ± 10 | -- | -- | -- | -- | -- |
| **Catechol** | 61.7 ± 0.7 | -- | 44 ± 38 | -- | 154 ± 1.0 | -- | 124 ± 19 | -- |
| **4-Methylcatechol** | 8.8 ± 0.1 | -- | 5.9 ± 5.1 | -- | 16.5 ± 1.0 | -- | 12.7 ± 1.4 | -- |
| **3-Methoxycatechol** | 17.0 ± 0.2 | -- | 11 ± 10 | -- | 17 ± 1.2 | -- | 9.9 ± 0.3 | -- |
| **Acetic acid** | 1786.8 ± 5.8 | -- | 1630.9 ± 4.1 | 10.5 ± 1.9 | 1669 ± 12 | -- | 1838 ± 36 | -- |
| **Formic acid** | 204.0 ± 7.8 | 90.0 ± 1.3 | 98.6 ± 2.4 | 49.0 ± 0.7 | 73.2 ± 1.5 | 28.8 ± 1.4 | 303.4 ± 6.7 | 104.2 ± 5.3 |
| **Glycolic acid** | 264 ± 14 | 99.4 ± 2.3 | 2687.6 ± 9.6 | 111.0 ± 3.2 | 218.0 ± 0.3 | 164.9 ± 3.9 | 172.7 ± 8.8 | 18.4 ± 0.2 |
| **Lactic acid** | 858 ± 15 | -- | 991.2 ± 6.3 | -- | 791.4 ± 8.9 | -- | 566 ± 27 | -- |

**Table S7.** **Mass of components present in the alkaline liquor (AL) and in the main streams of the 11 depolymerization reactions from 250 g of the AL**. DAL_1_ (180 °C, 30 min), DAL_2_ (180 °C, 30 min, 4 bar O_2_), DAL_3_ (180 °C, 90 min), DAL_4_ (180 °C, 90 min, 4 bar O_2_), triplicate reactions at central point (DAL_5-7_) 240 °C, 60 min, 2 bar O_2_), DAL_8_ (300 °C, 30 min), DAL_9_ (300 °C, 30 min, 4 bar O_2_), DAL_10_ (300 °C, 90 min), and DAL_11_ (300 °C, 90 min, 4 bar O_2_).  n = 1.

| Mass (g) | **AL** | **DAL_1_** | **DAL_2_** | **DAL_3_** | **DAL_4_** | **DAL_5_** | **DAL_6_** | **DAL_7_** | **DAL_8_** | **DAL_9_** | **DAL_10_** | **DAL_11_** |
| --- | --- | --- | --- | --- | --- | --- | --- | --- | --- | --- | --- | --- |
| *^a^*Aromatic monomers | 0.18 | 0.06 | 0.05 | 0.07 | 0.08 | 0.21 | 0.19 | 0.17 | 0.34 | 0.30 | 0.25 | 0.33 |
| *^b^*Organic acids | 0.88 | 0.99 | 0.76 | 0.92 | 1.08 | 1.34 | 1.25 | 1.30 | 1.07 | 1.36 | 1.02 | 1.44 |
| *^c^*Carbohydrates | 0.87*^e^* | 0.02 | 0.03 | 0.04 | 0.01 | 0.03 | 0.05 | 0.05 | 0.04 | 0.03 | 0.01 | 0.02 |
| *^d^*Solid fraction | 2.53 | 2.53 | 3.42 | 2.00 | 2.05 | 1.36 | 1.47 | 1.95 | 0.65 | 1.10 | 1.04 | 1.03 |

*^a^*Quantified by GC-MS. *^b^*Quantified by HPLC. *^c^*Arabinose, xylose and glucose, quantified by HPLC. *^d^*The solid fraction obtained after pH adjustment (1.0) and subsequent filtration is composed of hydrochar, ashes, and non-depolymerized lignin. *^e^*These sugars are not in monomeric form, they are in the form of xylan and glucan oligomers.

**Table S8. Concentration of aromatic compounds and organic acids measured during anaerobic reactions.***t_i_*: supernatant collected immediately after inoculation; *t_f_*: supernatant collected after 120 days of reaction. Total aromatics were determined by UV absorbance at 280 nm. Aromatic monomers were quantified by GC-MS, and aliphatic acids by HPLC. No sugars (glucose, xylose, arabinose) were detected. *nd*: not determined, indicates either consumption or absence in the initial sample (*t_i_*). *AL*: alkaline liquor from sugarcane bagasse. *DAL*: depolymerized alkaline liquor. *DAL_1_*: 180 °C, 30 min, 0 bar O₂; *DAL_5_*: 240 °C, 60 min, 2 bar O₂; *DAL_11_*: 300 °C, 90 min, 4 bar O₂. “--”: below detection limit or not detected. Data are shown as mean ± SD for t_f_ samples (n=3). For t_i_ samples (n=1).

| **Compounds (μg mL^-1^)** | **AL** | | **DAL_1_** | | | | **DAL_5_** | | | **DAL_11_** | |
| --- | --- | --- | --- | --- | --- | --- | --- | --- | --- | --- | --- |
|  | **t*_i_*** | **t*_f_*** | | **t*_i_*** | **t*_f_*** | **t*_i_*** | | **t*_f_*** | **t*_i_*** | | **t*_f_*** |
| **Total aromatics (UV-vis)** | 8522.4 | 3569 ± 420 | | 5653.2 | 2930 ± 326 | 6927.5 | | 4013 ± 452 | 1969.7 | | 1165 ± 92 |
| **Phenol** | 9.0 | -- | | 28.5 | 46.8 ± 7.5 | 111.1 | | 26 ± 37 | 270.9 | | 8.2 ± 1.1 |
| **4-Methylphenol** | -- | -- | | -- | 24.2 ± 6.8 | -- | | 5.8 ± 5.0 | 9.2 | | 30 ± 20 |
| **4-Ethylphenol** | -- | 15 ± 12 | | 2.0 | 8.7 ± 0.9 | 2.0 | | 5.4 ± 3.0 | 8.2 | | 7.6 ± 1.4 |
| **4-Hydroxybenzaldehyde** | -- | -- | | 2.0 | -- | 5.6 | | -- | -- | | -- |
| **4-Coumaric acid** | 541.0 | -- | | -- | -- | -- | | -- | -- | | -- |
| **Guaiacol** | 4.8 | -- | | 10.7 | -- | 33.0 | | -- | 14.4 | | -- |
| **Ferulic acid** | 24.2 | -- | | -- | -- | -- | | -- | -- | | -- |
| **Syringol** | -- | -- | | -- | -- | 5.5 | | -- | -- | | -- |
| **Acetosyringone** | -- | -- | | 3.9 | -- | 9.4 | | -- | -- | | -- |
| **Syringaldehyde** | -- | -- | | -- | -- | 3.7 | | -- | -- | | -- |
| **Catechol** | -- | -- | | -- | 9.5 ± 1.5 | -- | | -- | -- | | -- |
| **Formic acid** | -- | -- | | 35.2 | -- | 88.6 | | -- | 39.0 | | -- |
| **Acetic acid** | 1893.5 | -- | | 2661.7 | 188 ± 136 | 2772.1 | | -- | 3465.7 | | 36 ± 22 |
| **Glycolic acid** | 54.4 | -- | | 137.5 | -- | 619.1 | | 168 ± 291 | 665.4 | | -- |
| **Lactic acid** | 36.7 | -- | | 318.6 | -- | 752.1 | | -- | 897.2 | | -- |
